# Supplementary material for: 5,5,5-Trichloropent-3-en-one as a Precursor of 1,3-Bi-centered Electrophile in Reactions with Arenes in Brønsted Superacid CF3SO3H. Synthesis of 3-Methyl-1-trichloromethylindenes
Source: Molecules. 2022 Oct 7;27(19):6675. doi: 10.3390/molecules27196675 (PMC9573653; doi:10.3390/molecules27196675)
Supplement: Supplementary file 1 [file molecules-27-06675-s001.zip › molecules-1935676-supplementary.pdf]

Supporting Information

5,5,5-Trichloropent-3-en-one as a Precursor of 1,3-Bi-centered Electrophile in Reactions with Arenes in Brønsted Superacid CF_3SO_3H. Synthesis of 3-Methyl-1-trichloromethylindenes

Ivan A. Shershnev [1], Irina A. Boyarskaya [1] and Aleksander V. Vasilyev [1,2,*]

[1] Department of Organic Chemistry, Institute of Chemistry, Saint Petersburg State University, Universitetskaya nab., 7/9, Saint Petersburg 199034, Russia
[2] Department of Chemistry, Saint Petersburg State Forest Technical University, Institutsky per., 5, Saint Petersburg 194021, Russia
* Correspondence: aleksvasil@mail.ru or a.vasilyev@spbu.ru

Contents

1. ^{1}H and ^{13}C NMR spectra of compounds **1, 2a-f, 3, 4a,b,** and cation **A** in Brønsted acids………S2

2. Data of DFT calculations of compound **1**, and cations **A, B1, C**………………………………S16

1. ^{1}H and ^{13}C NMR spectra of compounds 1, 2a-f, 3, 4a,b, and cation A in Brønsted acids

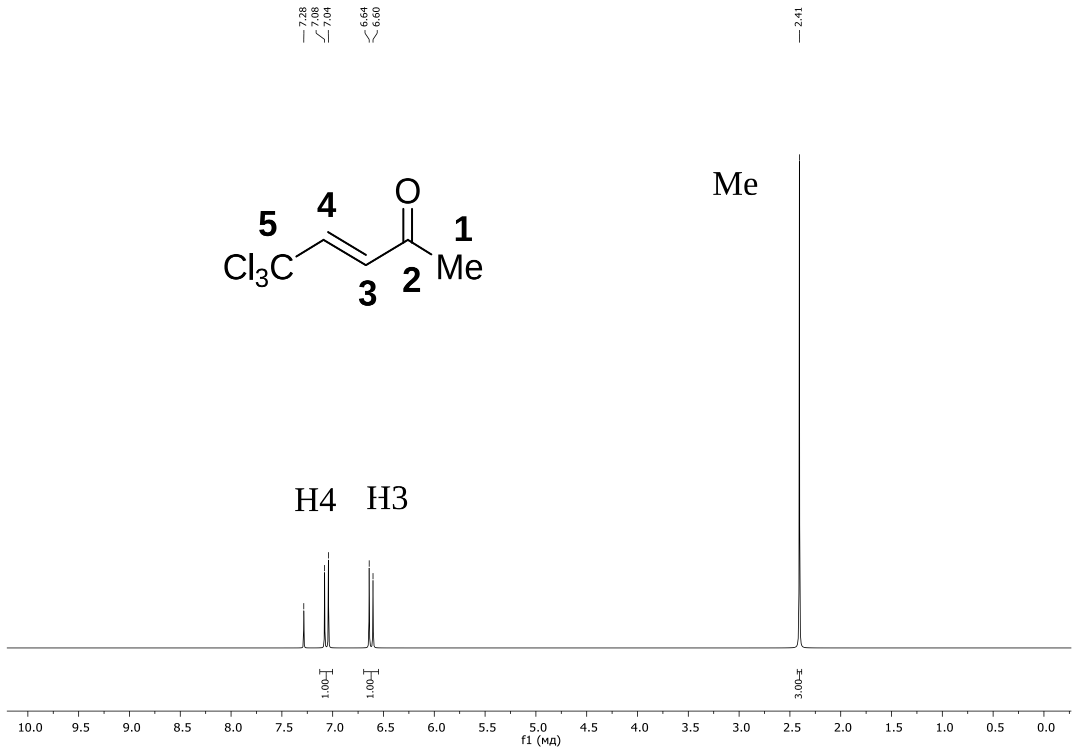

Figure S1. ^{1}H NMR spectrum of the compound **1** ($CDCl_3$, 400 MHz).

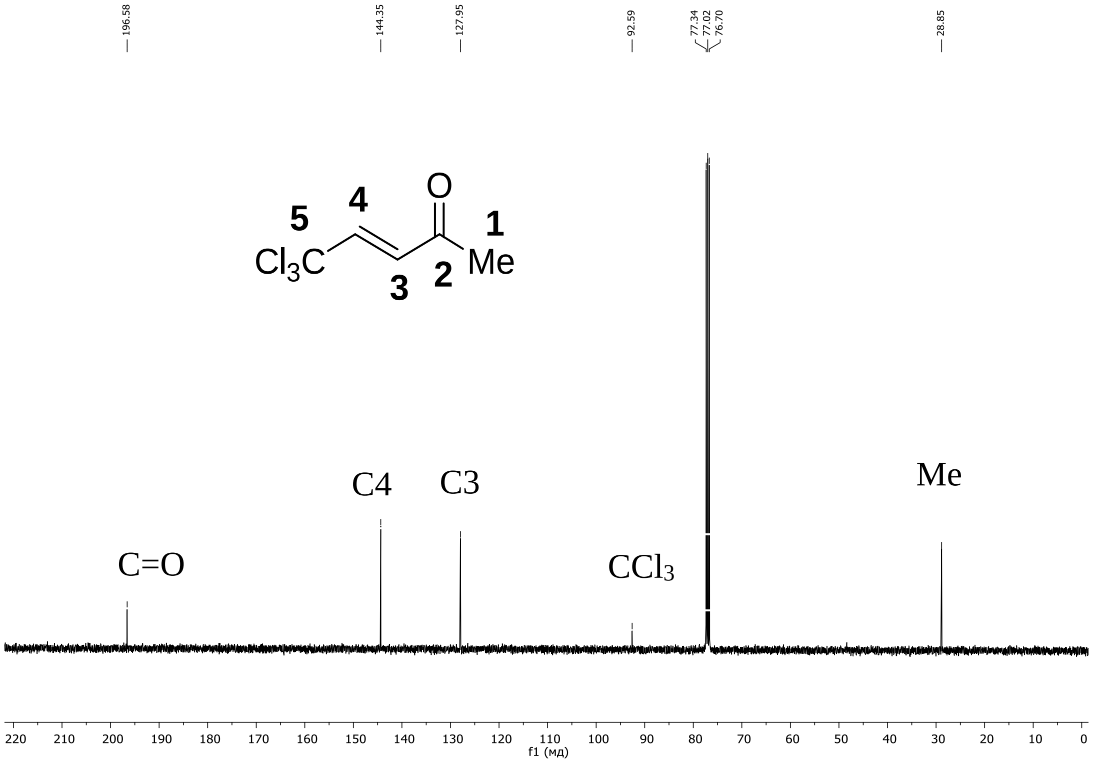

Figure S2. ^{13}C NMR spectrum of the compound **1** ($CDCl_3$, 101 MHz).

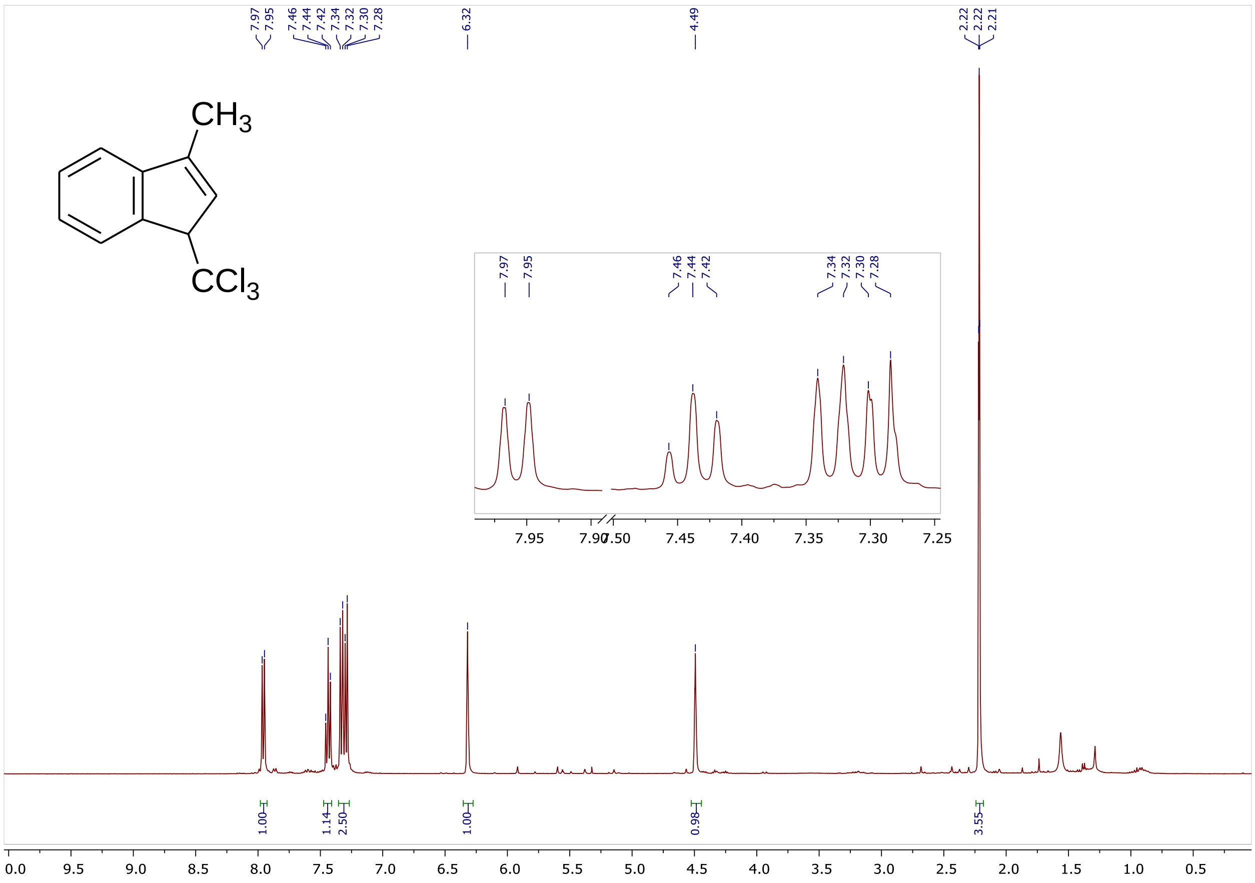

Figure S3. ^{1}H NMR spectrum of the compound **2a** ($CDCl_3$, 400 MHz).

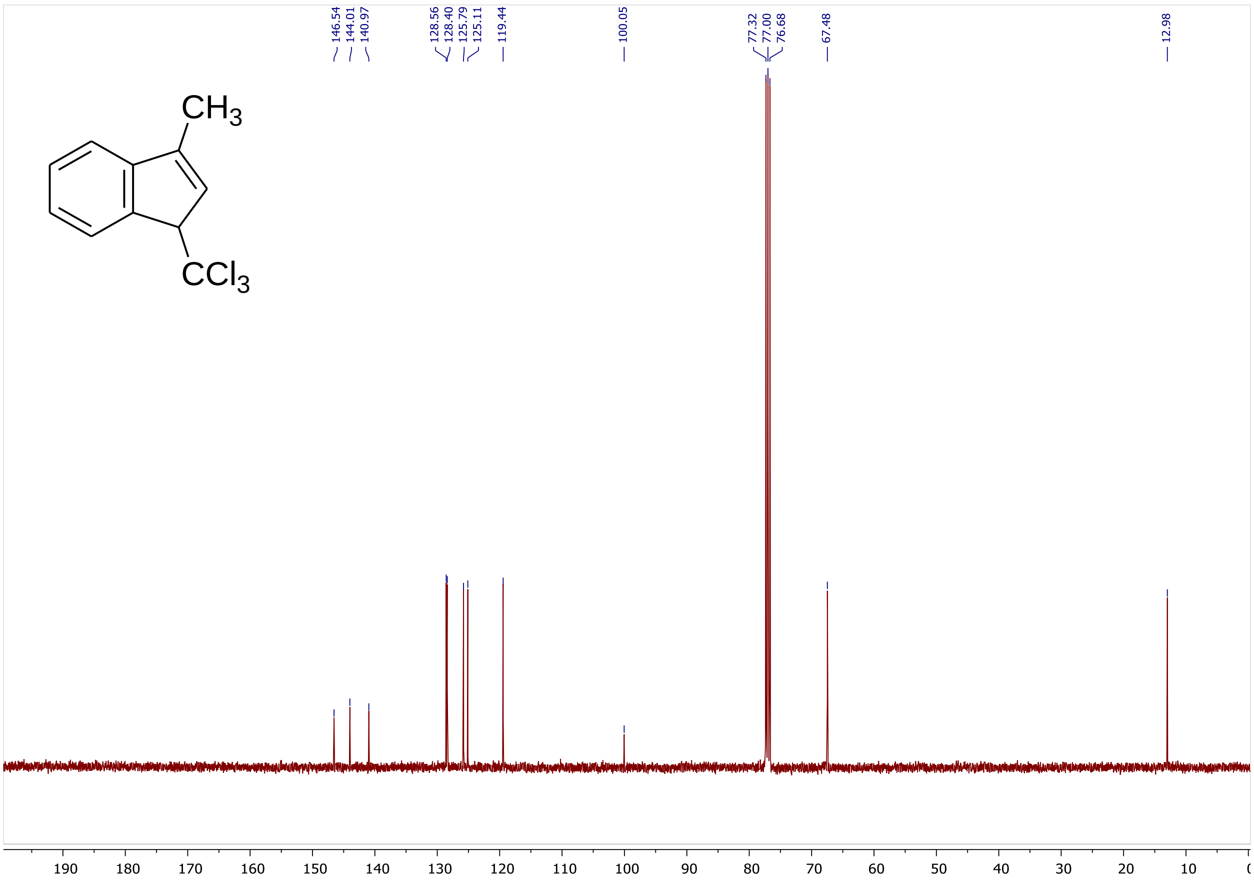

Figure S4. ^{13}C NMR spectrum of the compound **2a** ($CDCl_3$, 101 MHz).

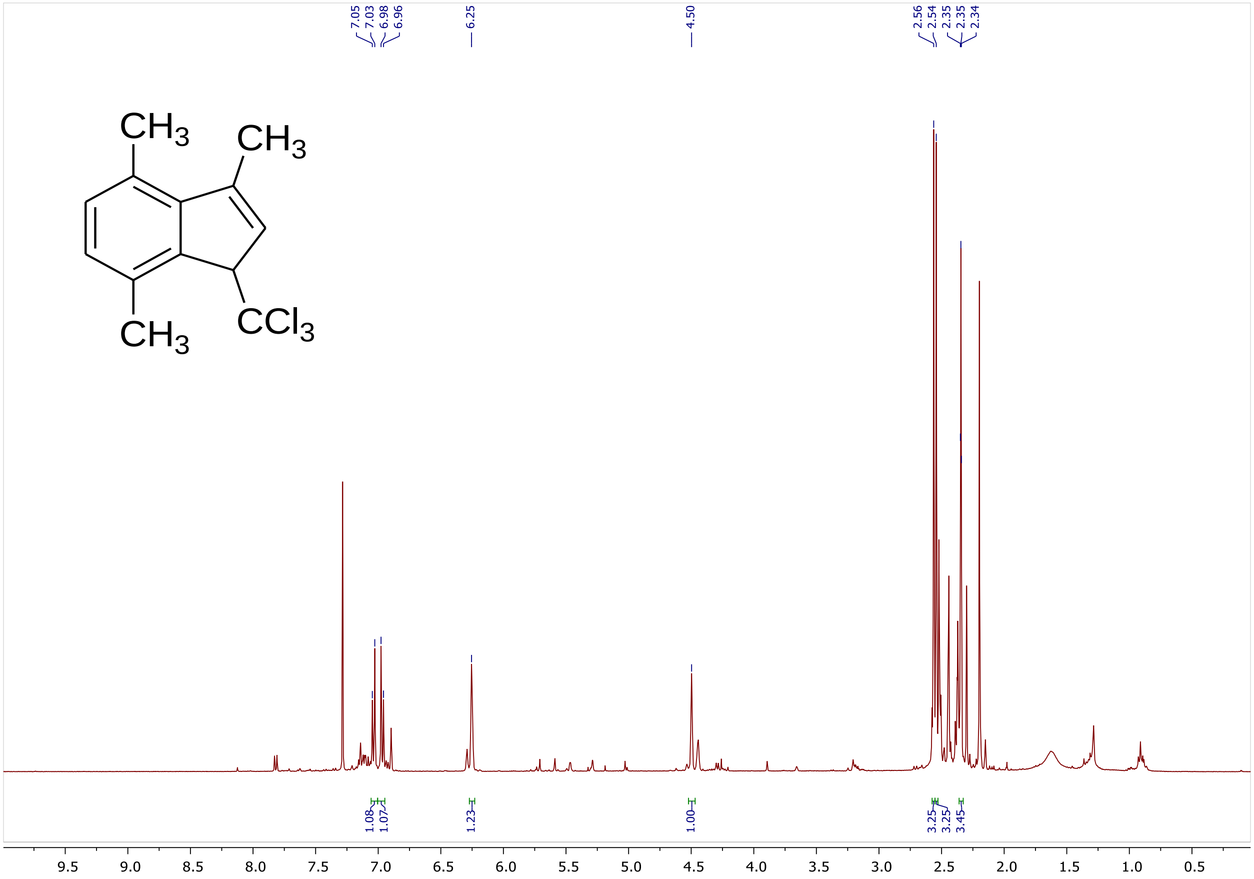

Figure S5. ^{1}H NMR spectrum of the compound **2b** ($CDCl_3$, 400 MHz).

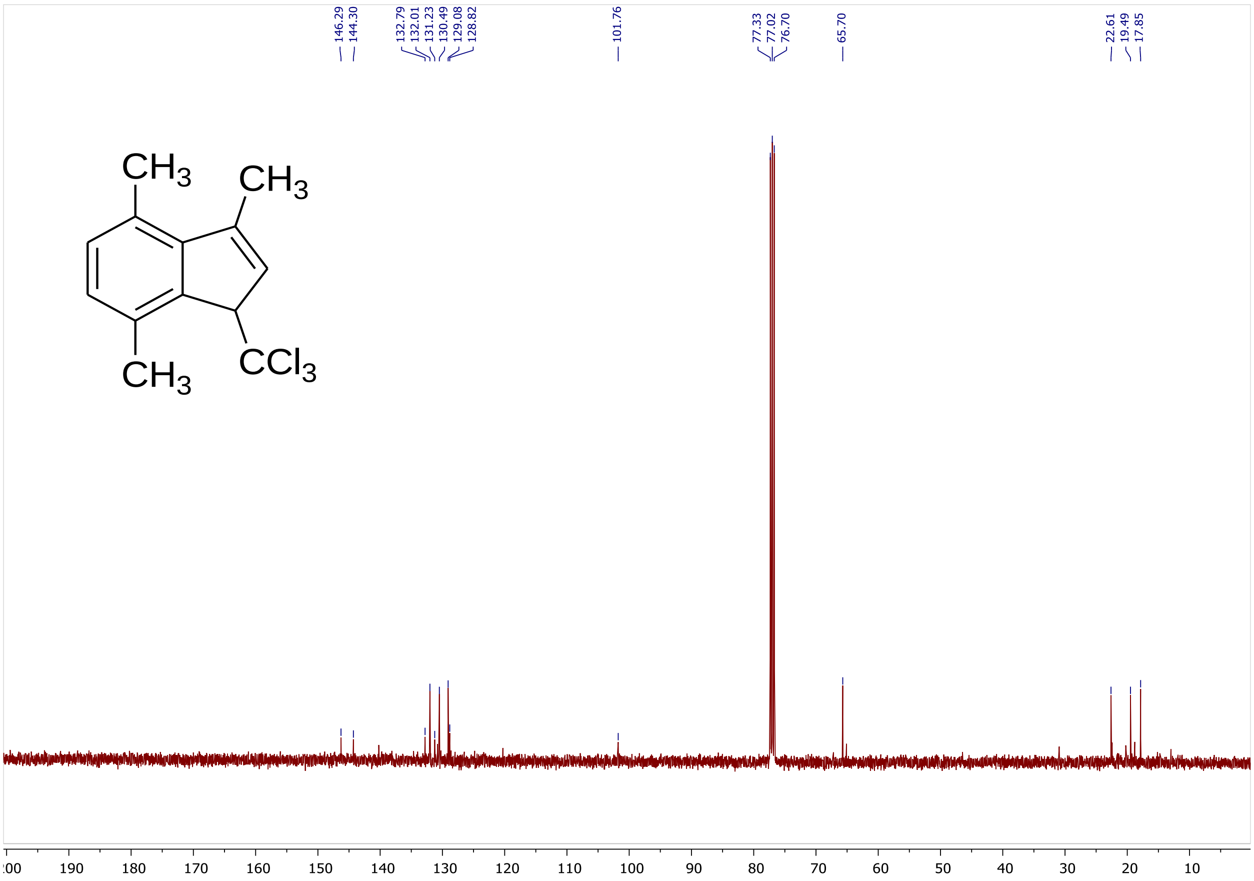

Figure S6. ^{13}C NMR spectrum of the compound **2b** ($CDCl_3$, 101 MHz).

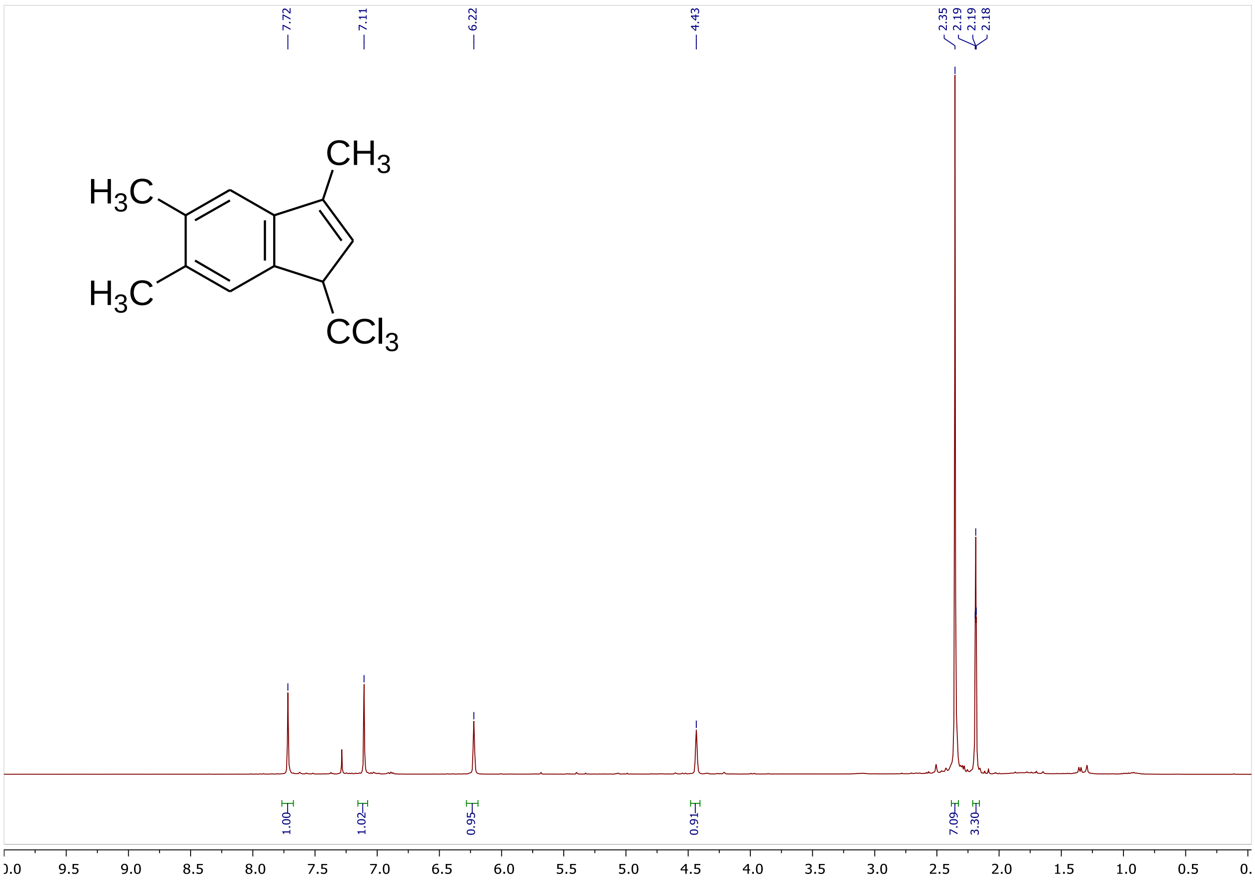

Figure S7. ^{1}H NMR spectrum of the compound **2c** ($CDCl_3$, 400 MHz).

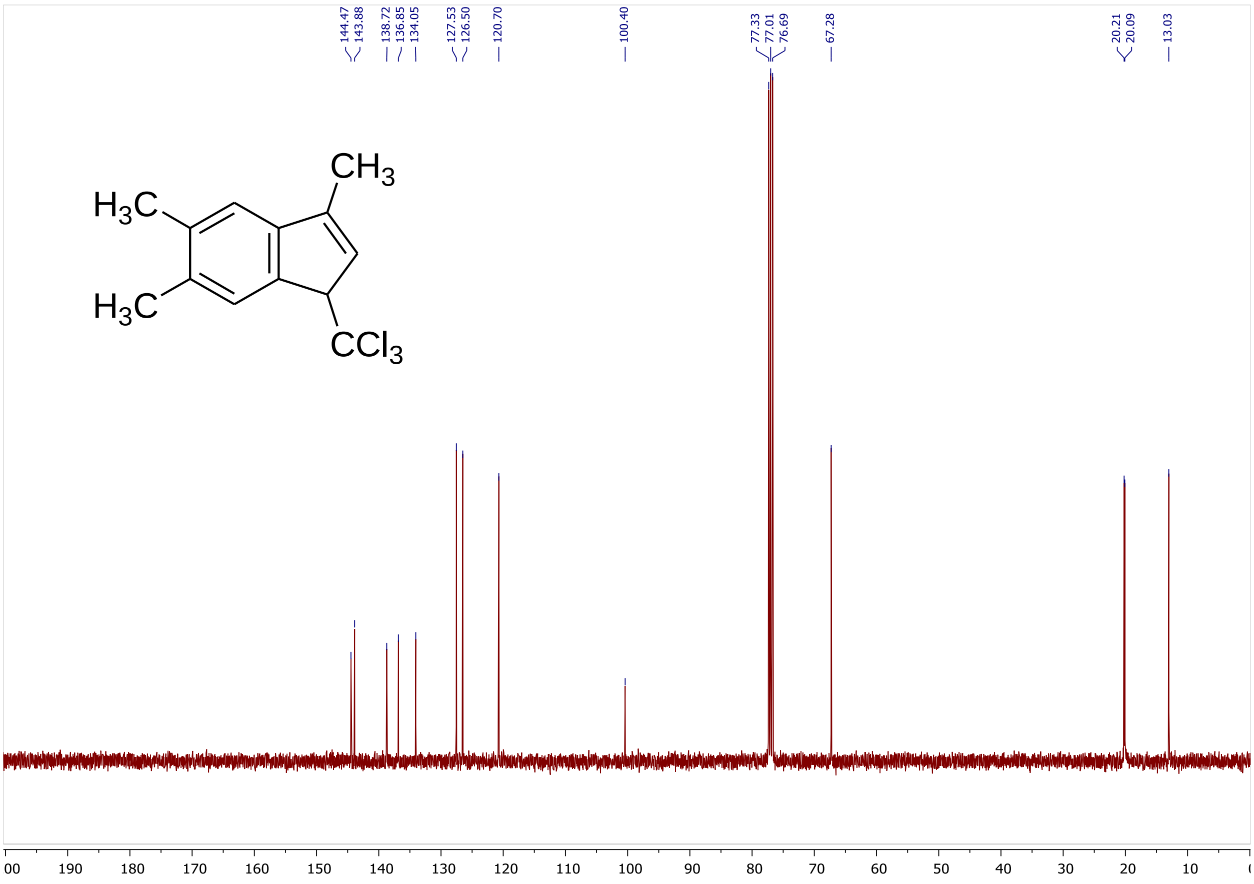

Figure S8. ^{13}C NMR spectrum of the compound **2c** ($CDCl_3$, 101 MHz).

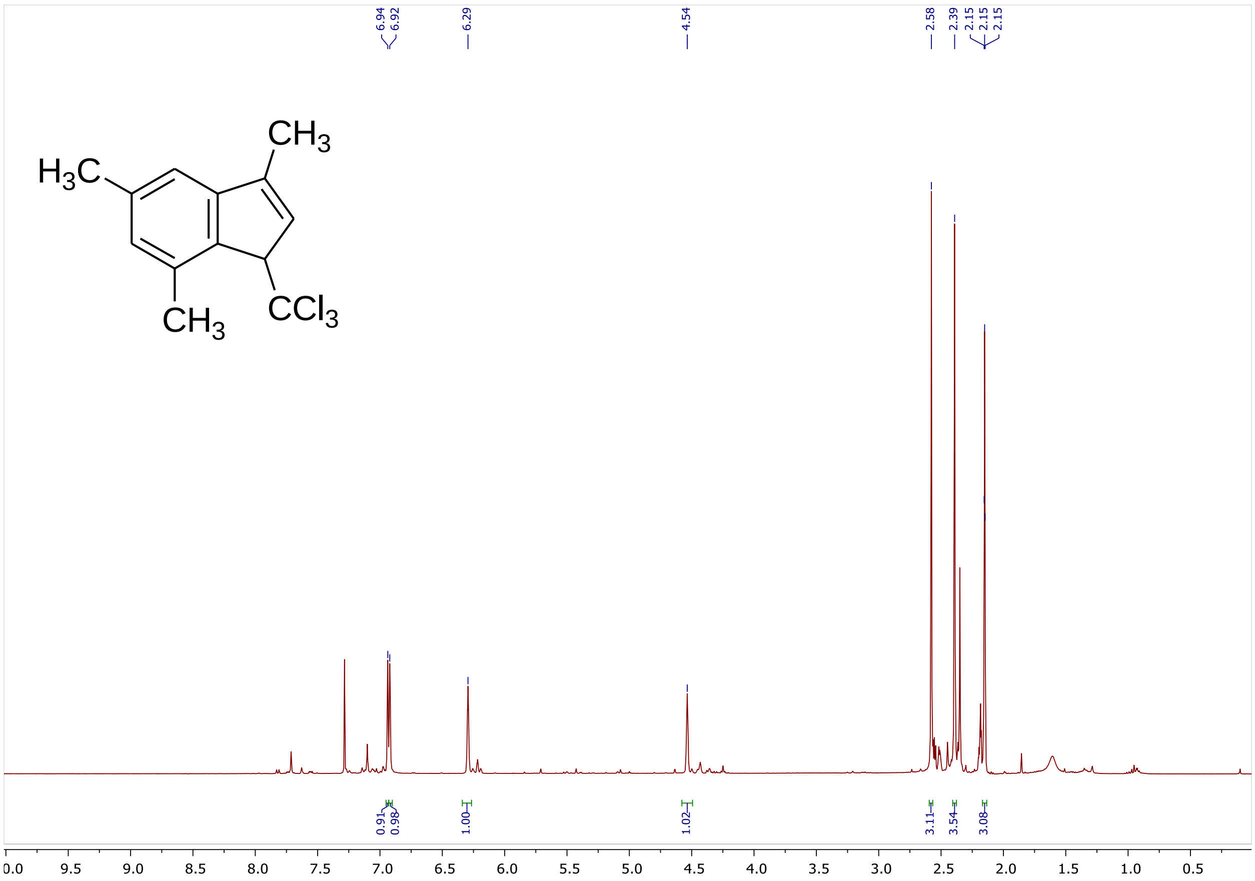

Figure S9. ^{1}H NMR spectrum of the compound **2d** ($CDCl_3$, 400 MHz).

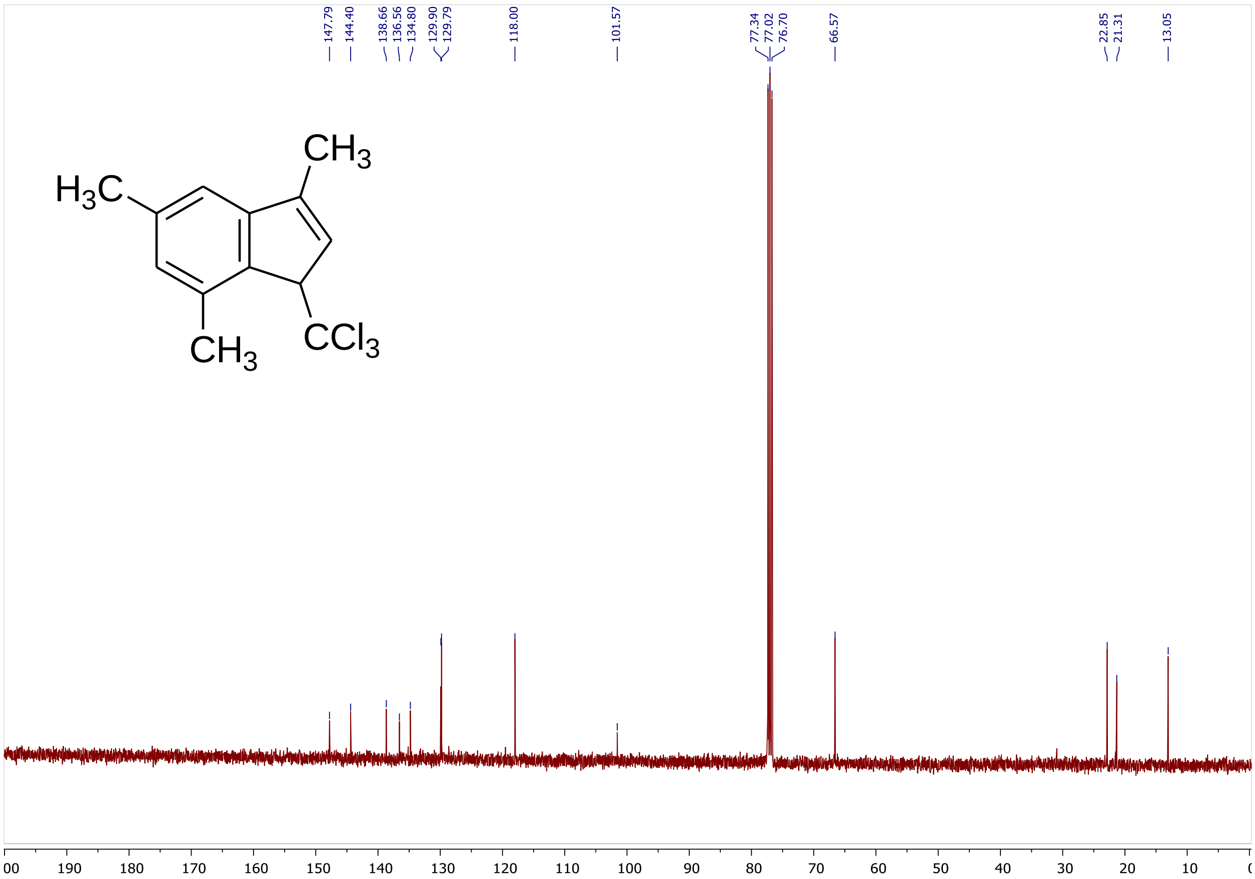

Figure S10. ^{13}C NMR spectrum of the compound **2d** ($CDCl_3$, 101 MHz).

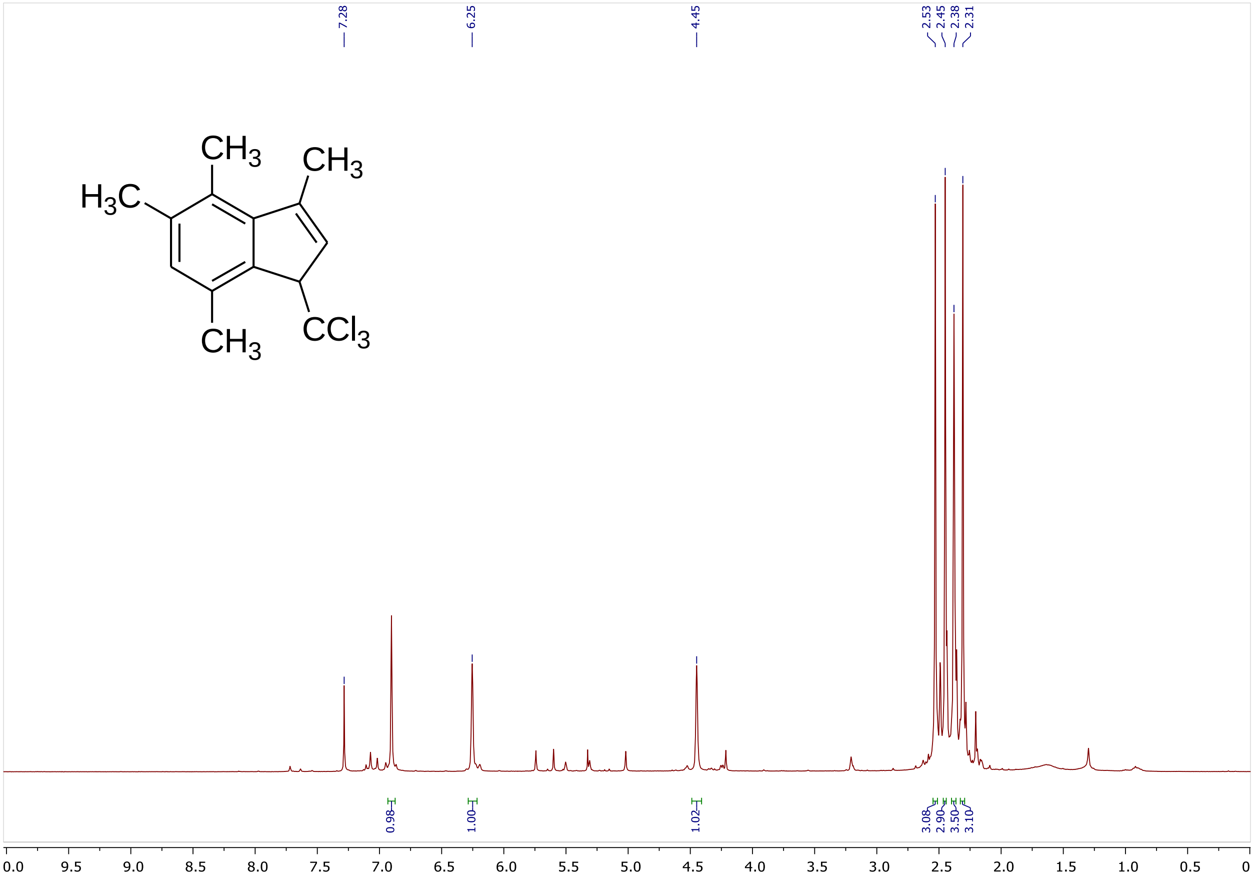

Figure S11. ^{1}H NMR spectrum of the compound **2e** ($CDCl_3$, 400 MHz).

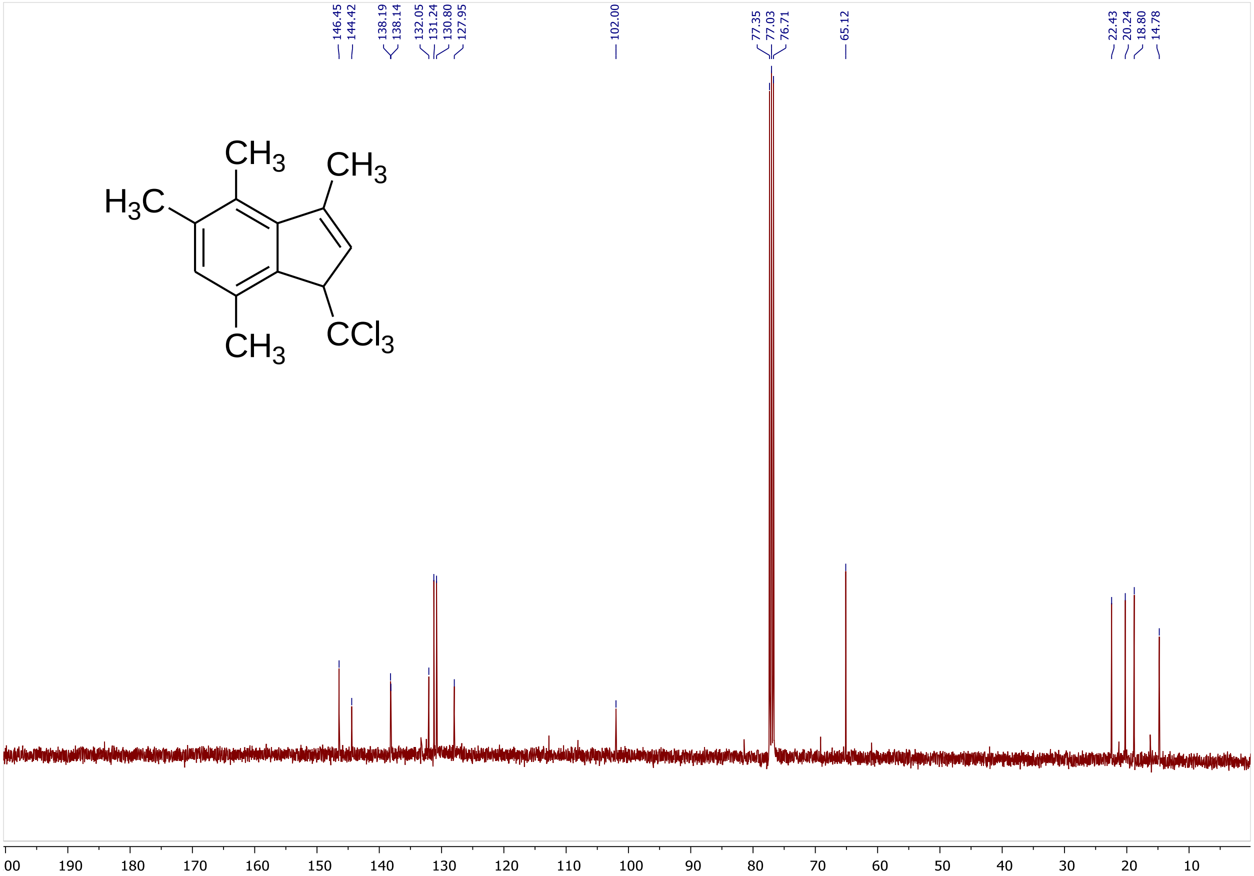

Figure S12. ^{13}C NMR spectrum of the compound **2e** ($CDCl_3$, 101 MHz).

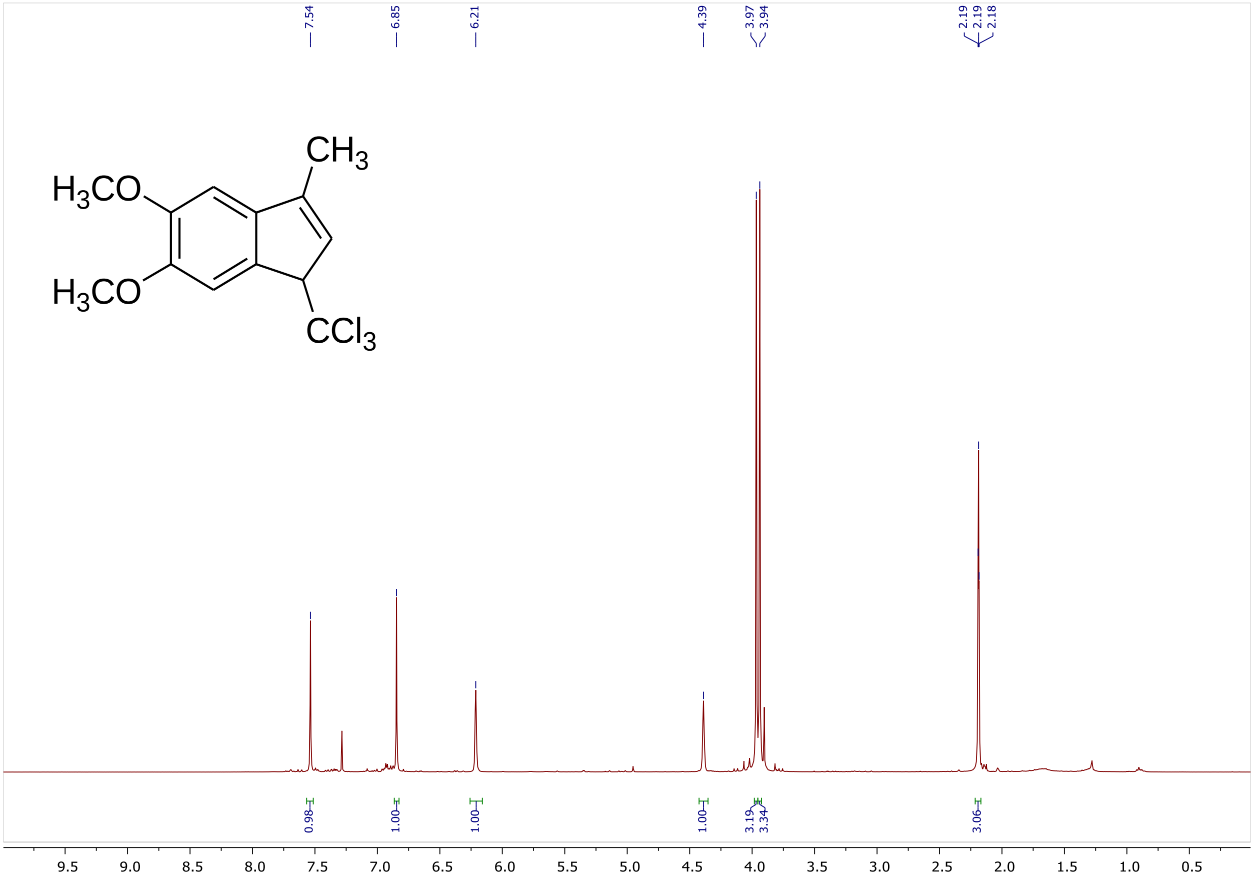

Figure S13. ^{1}H NMR spectrum of the compound **2f** ($CDCl_3$, 400 MHz).

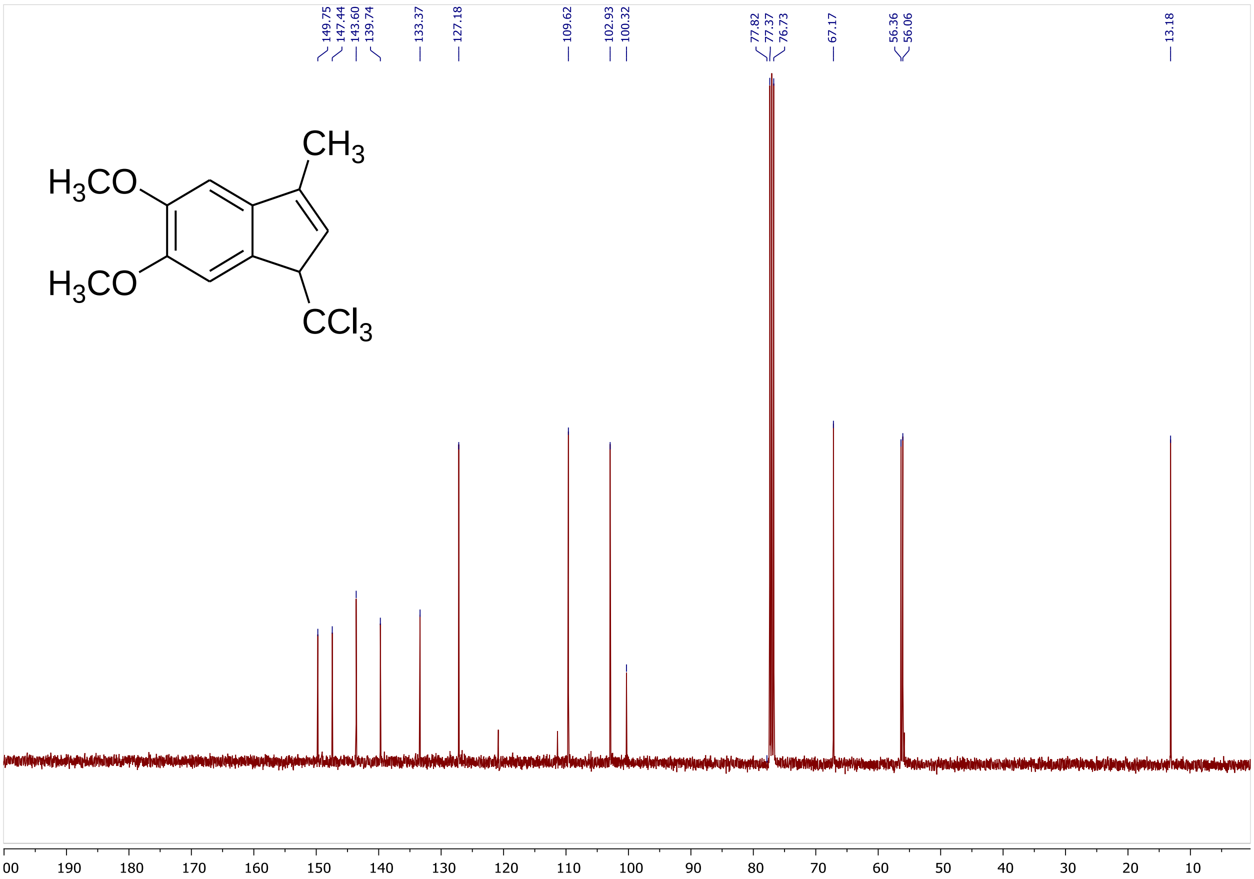

Figure S14. ^{13}C NMR spectrum of the compound **2f** ($CDCl_3$, 101 MHz).

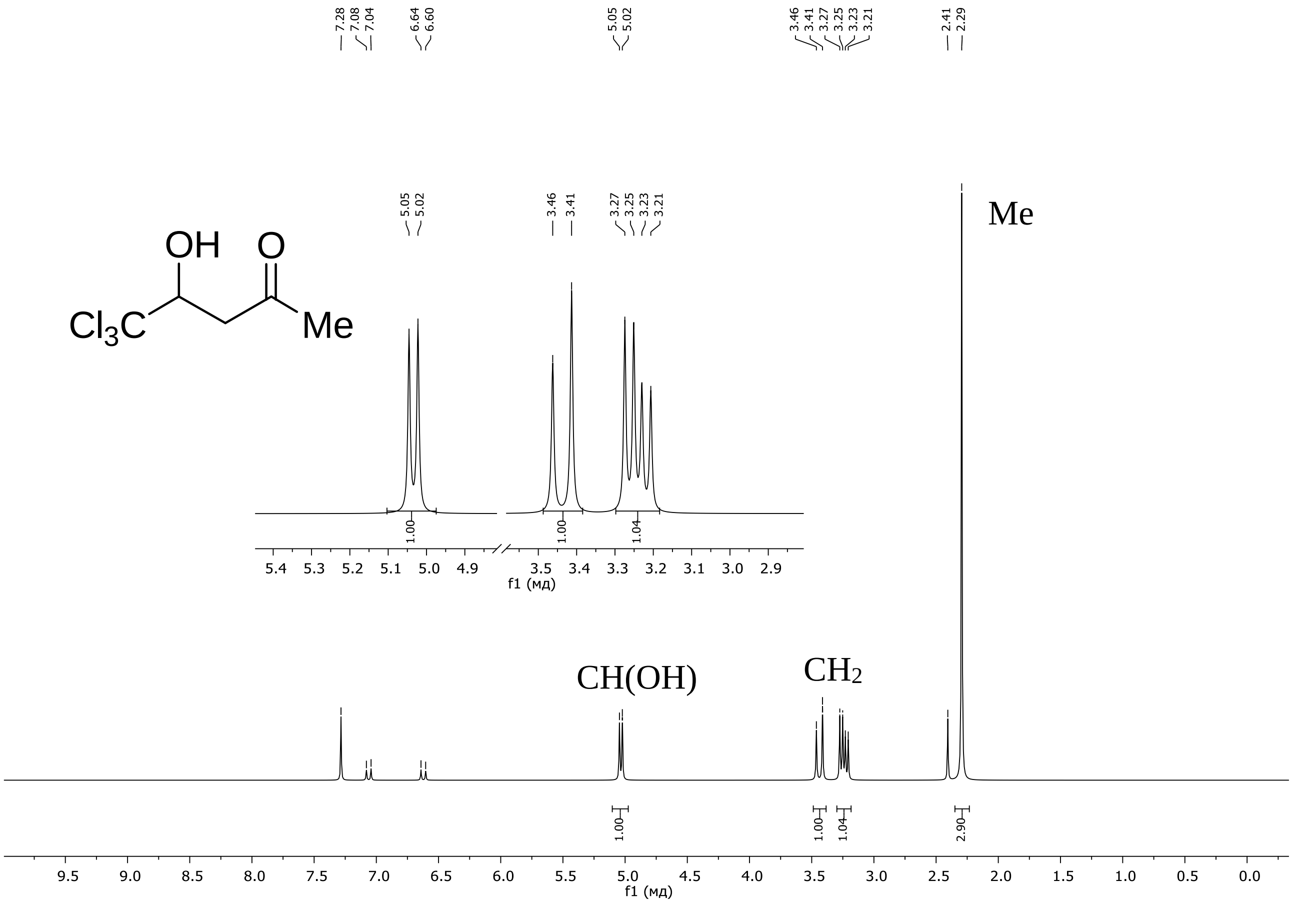

Figure S15. ^{1}H NMR spectrum of the compound **3** ($CDCl_3$, 400 MHz).

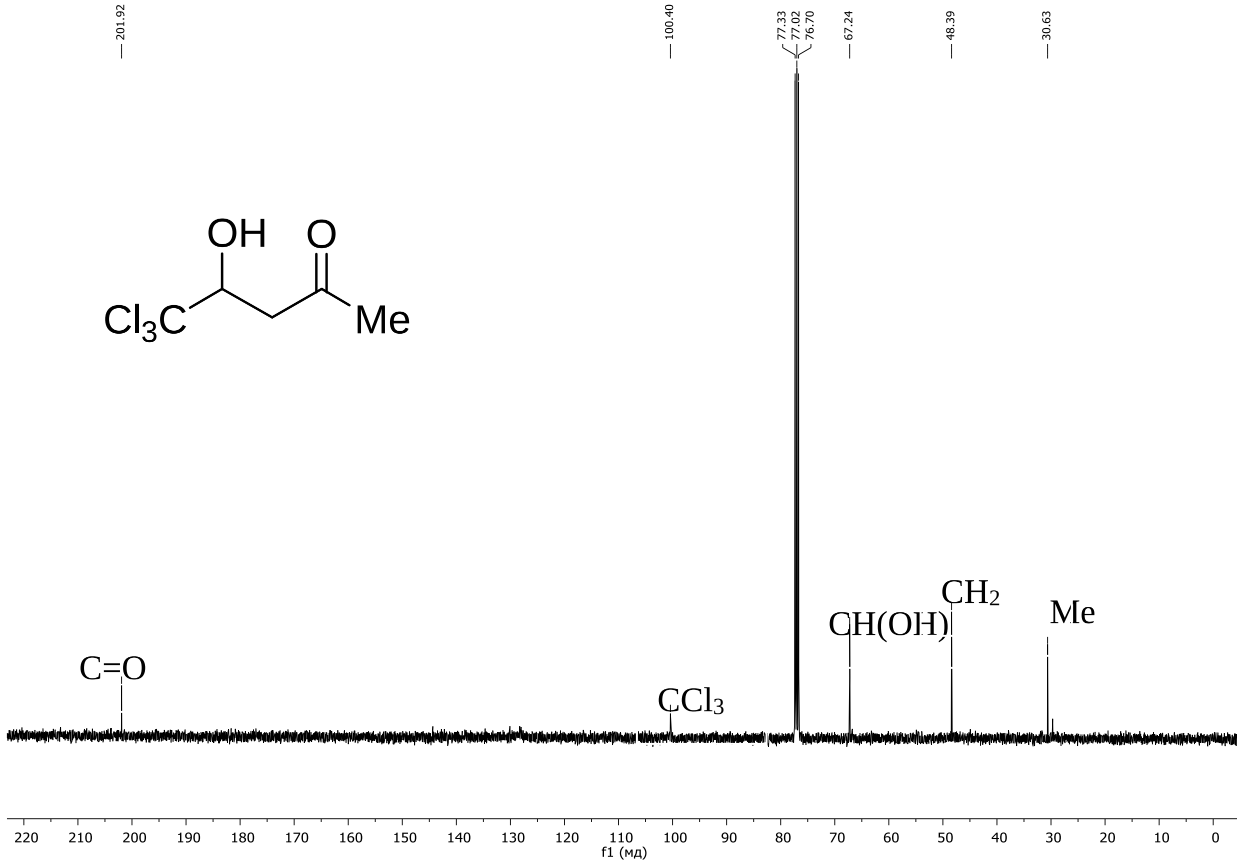

Figure S16. ^{13}C NMR spectrum of the compound **3** ($CDCl_3$, 101 MHz).

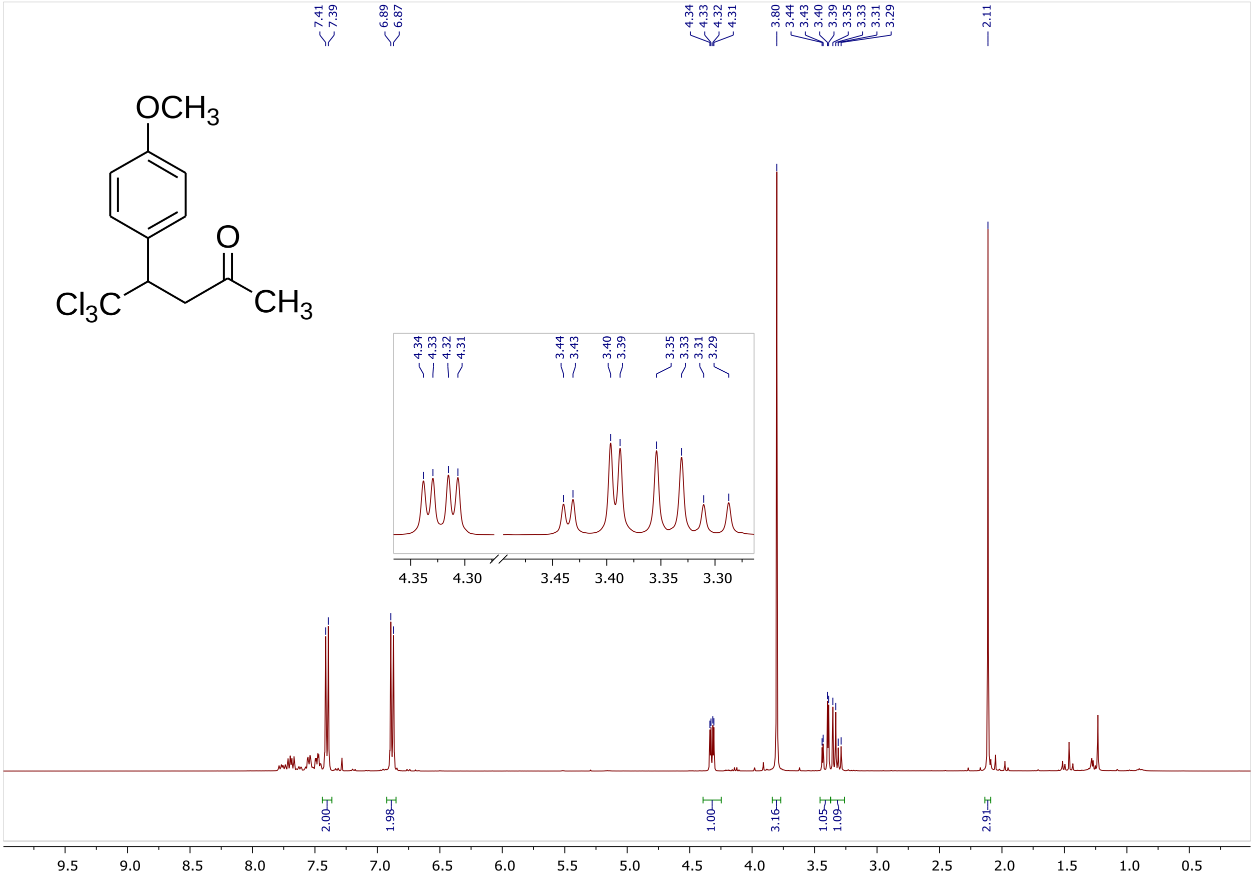

Figure S17. ^{1}H NMR spectrum of the compound **4a** ($CDCl_3$, 400 MHz).

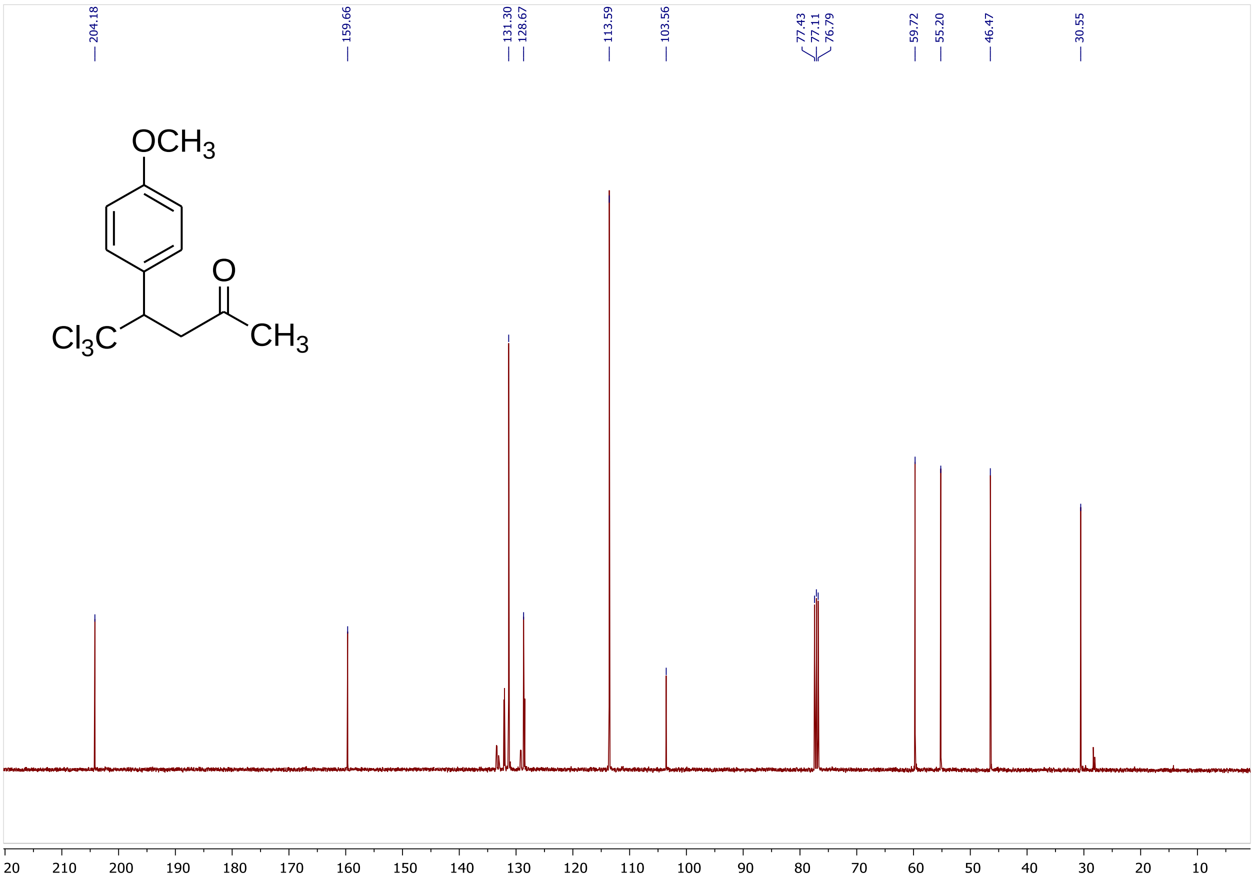

Figure S18. ^{13}C NMR spectrum of the compound **4a** ($CDCl_3$, 101 MHz).

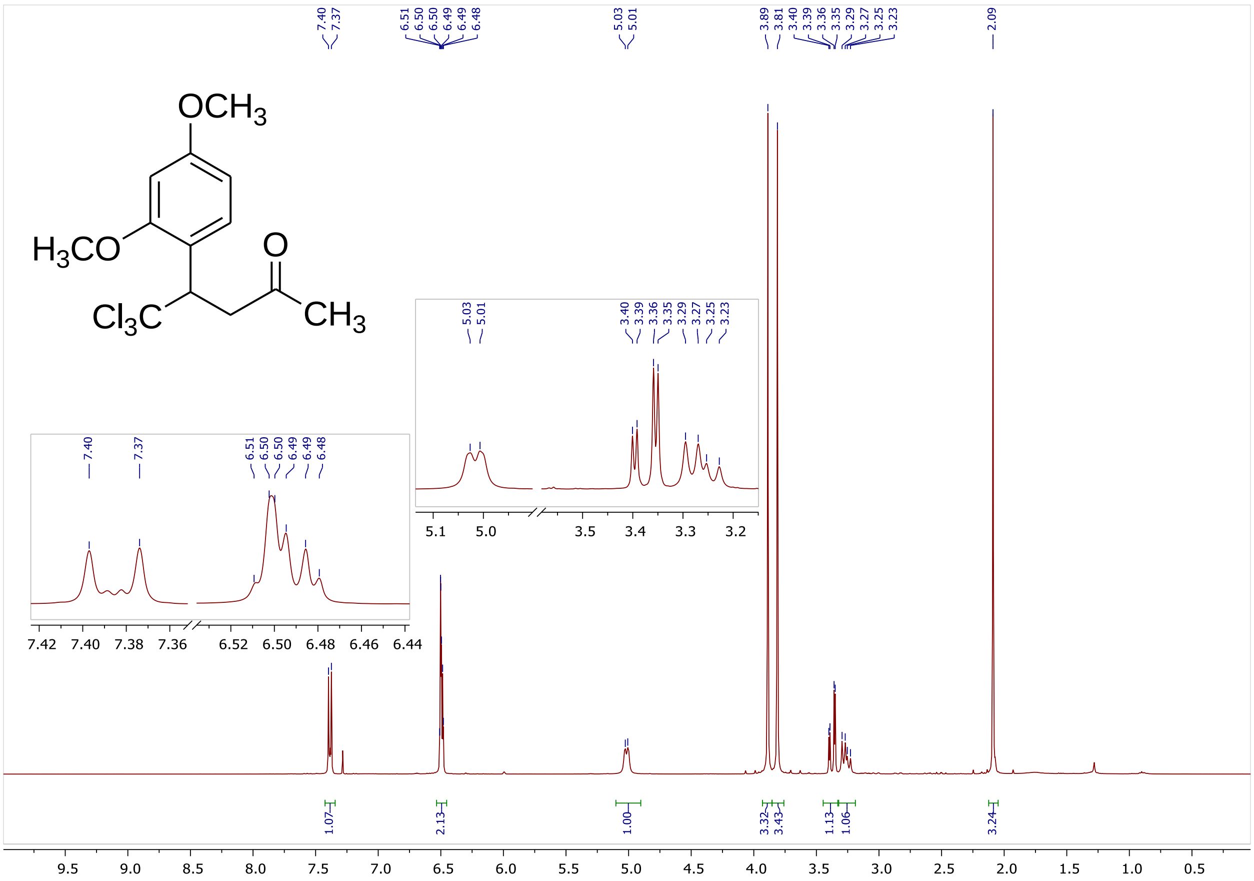

Figure S19. ^{1}H NMR spectrum of the compound **4b** ($CDCl_3$, 400 MHz).

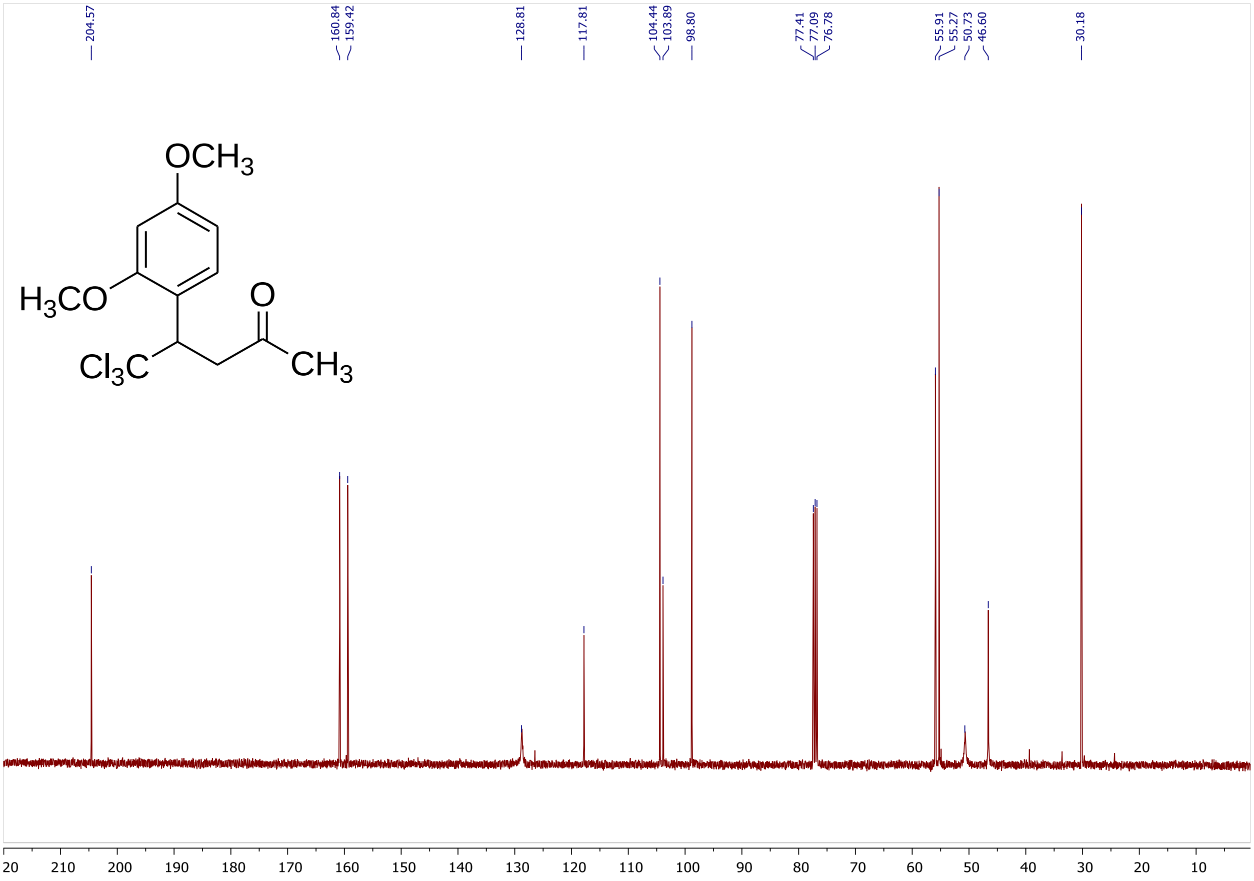

Figure S20. ^{13}C NMR spectrum of the compound **4b** ($CDCl_3$, 101 MHz).

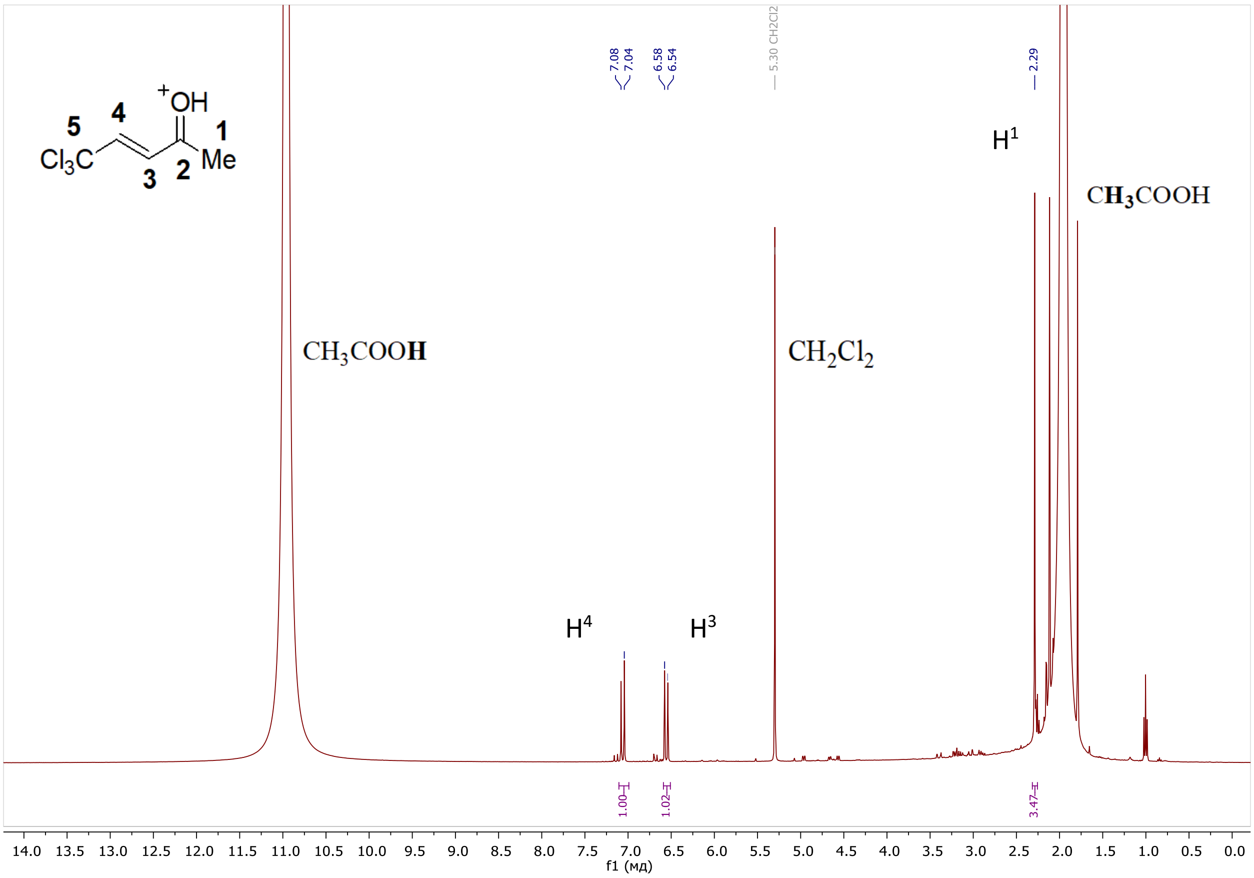

Figure S21. ^{1}H NMR spectrum of the cation **A** in CH_3COOH (CH_2Cl_2 as an internal standard, 400 MHz).

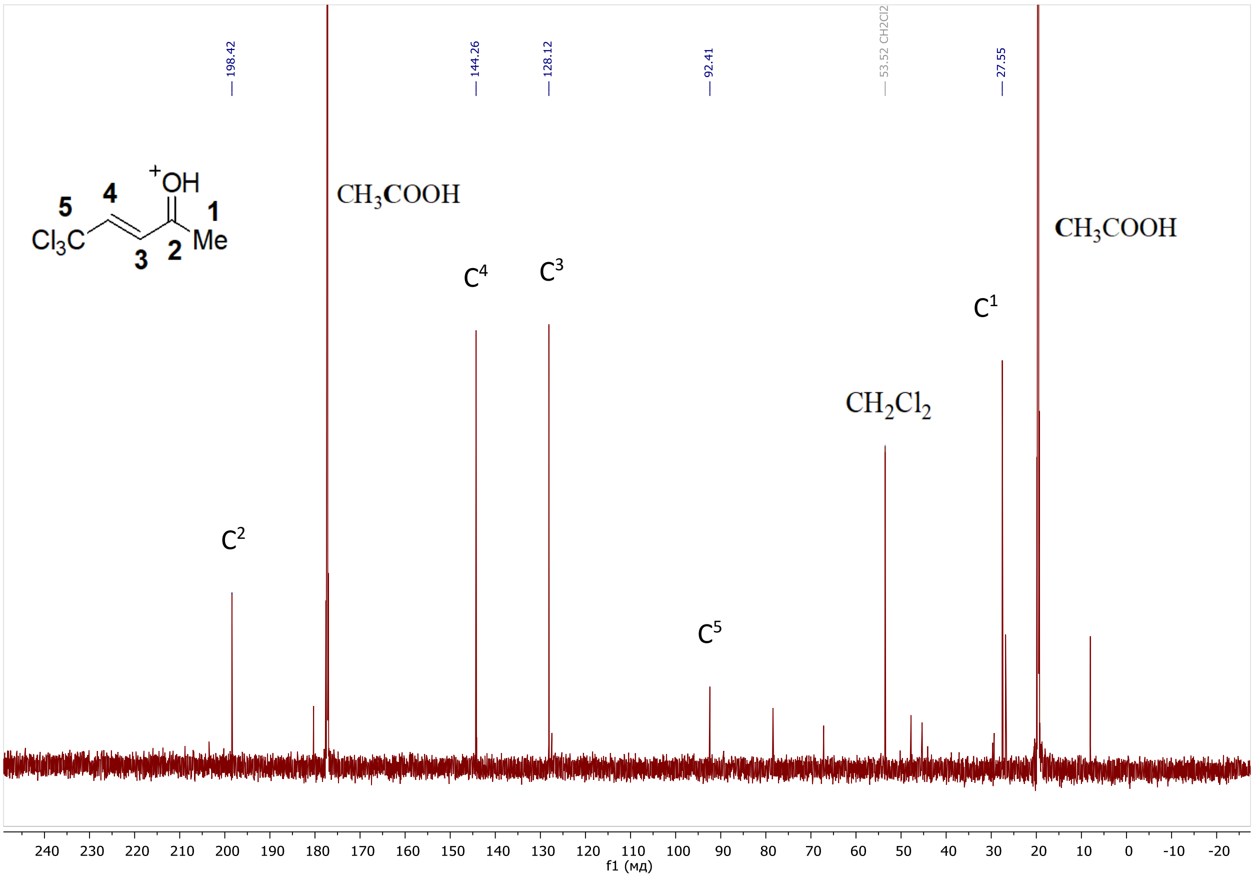

Figure S22. ^{13}C NMR spectrum of the cation **A** in CH_3COOH (CH_2Cl_2 as an internal standard, 101 MHz).

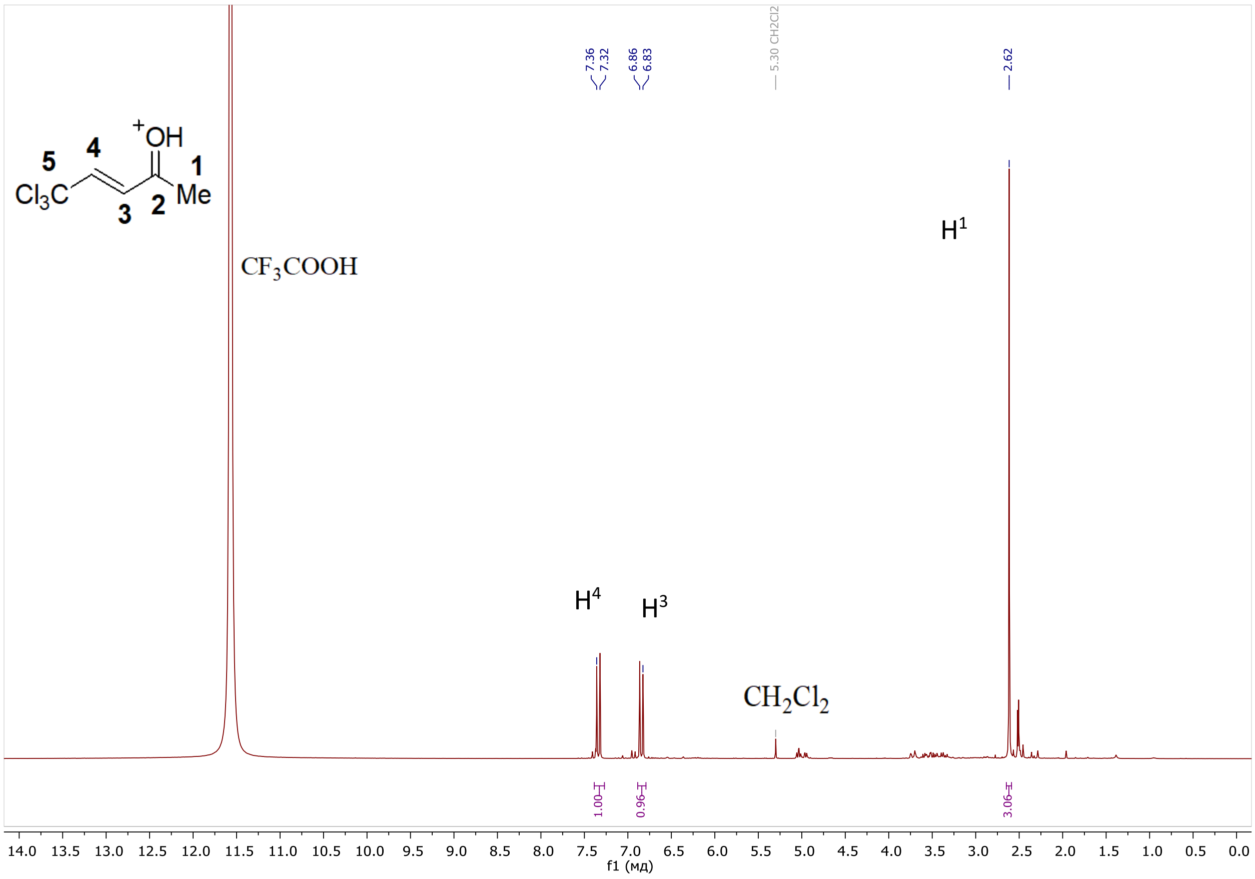

Figure S23. ^{1}H NMR spectrum of the cation **A** in CF_3COOH (CH_2Cl_2 as an internal standard, 400 MHz).

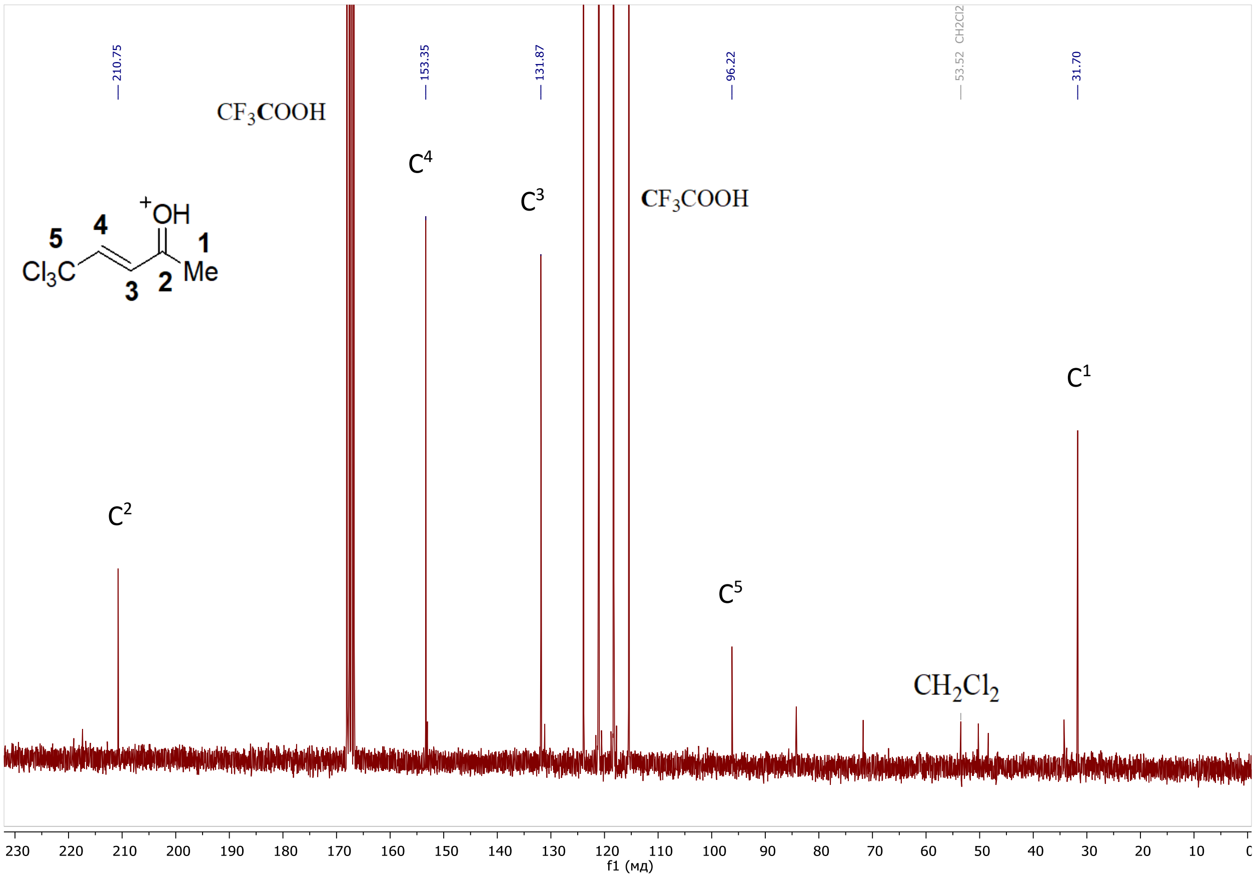

Figure S24. ^{13}C NMR spectrum of the cation **A** in CF_3COOH (CH_2Cl_2 as an internal standard, 101 MHz).

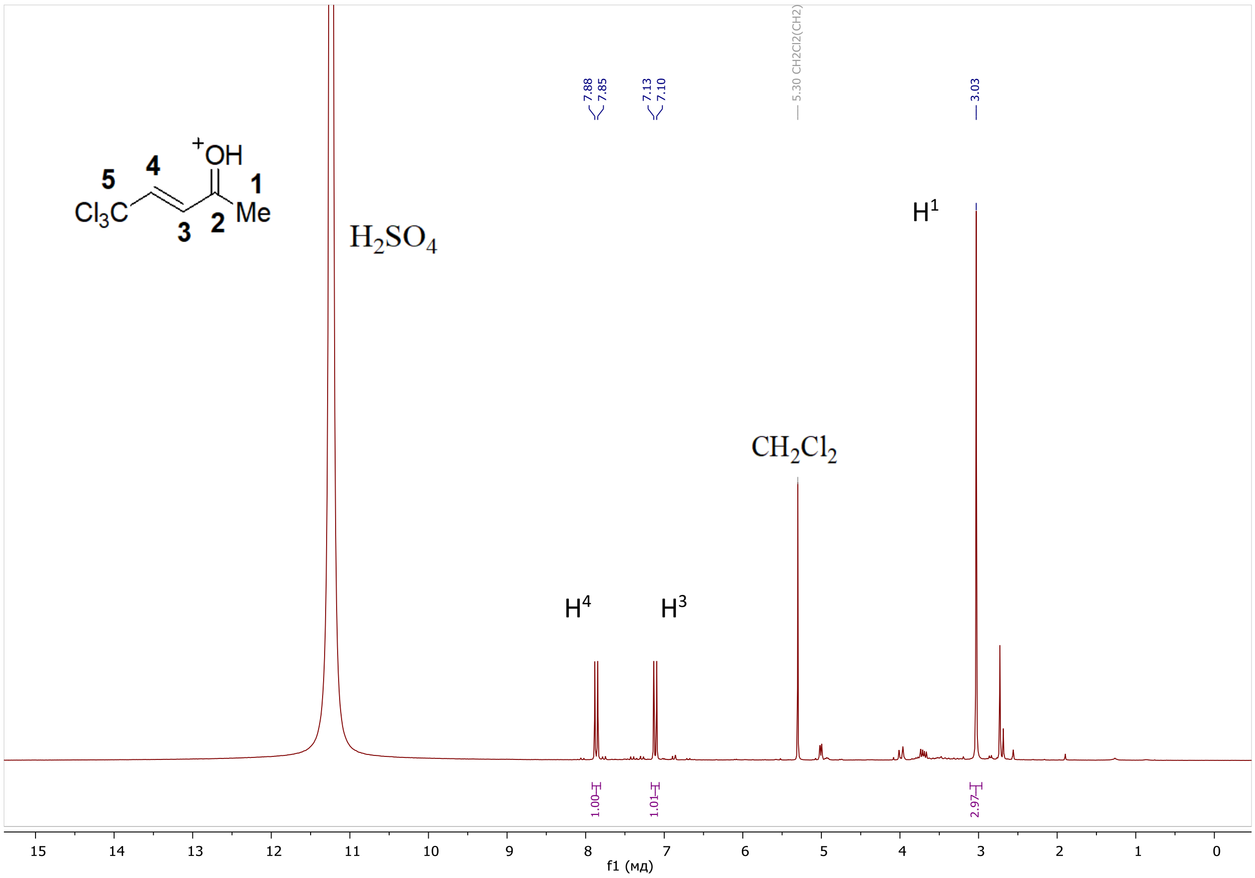

Figure S25. ^{1}H NMR spectrum of the cation **A** in H_2SO_4 (CH_2Cl_2 as an internal standard, 400 MHz).

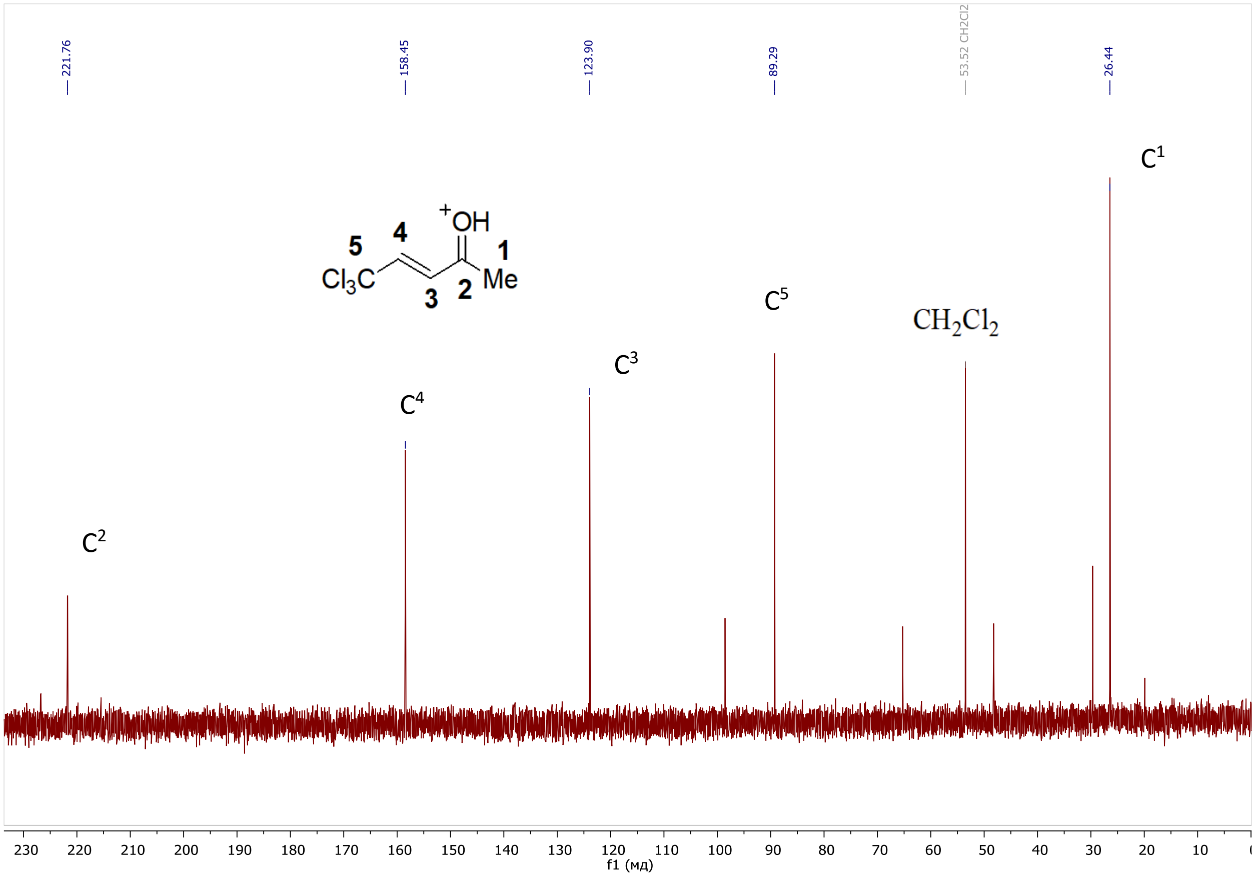

Figure S26. ^{13}C NMR spectrum of the cation **A** in H_2SO_4 (CH_2Cl_2 as an internal standard, 101 MHz).

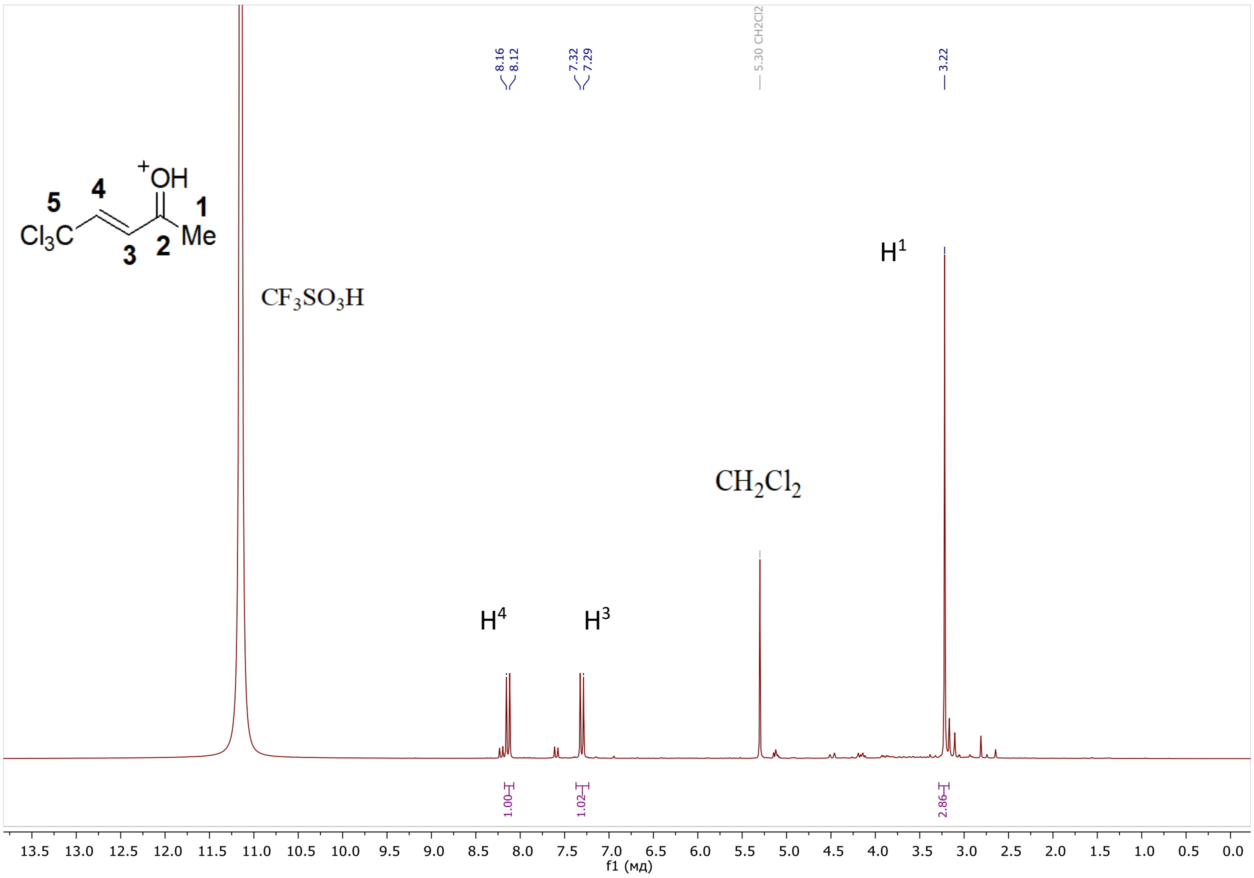

Figure S27. ^{1}H NMR spectrum of the cation **A** in CF_3SO_3H (CH_2Cl_2 as an internal standard, 400 MHz).

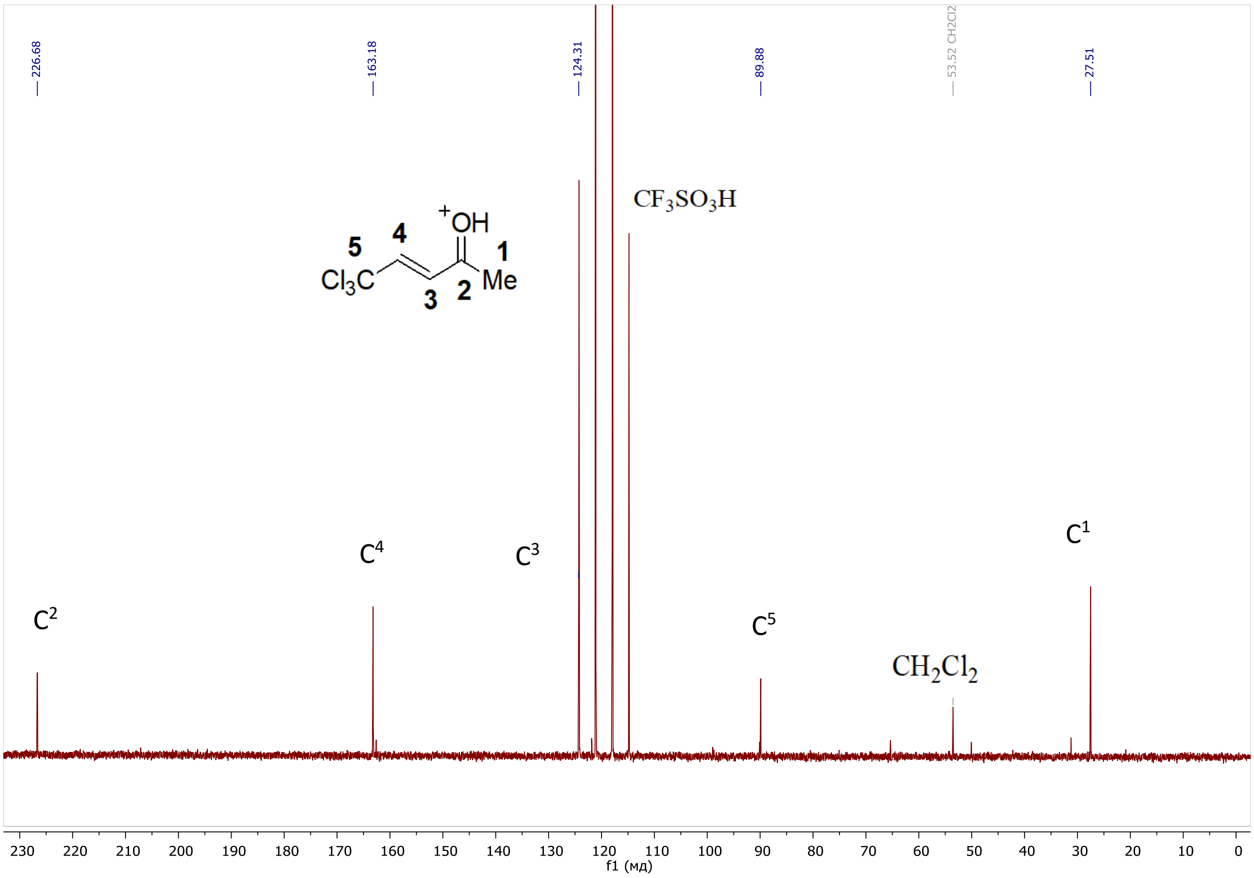

Figure S28. ^{13}C NMR spectrum of the cation **A** in CF_3SO_3H (CH_2Cl_2 as an internal standard, 101 MHz).

2. Data of DFT calculations of compound 1, and cations A, B1, C

Compound 1.
Energy E(B3LYP) = -1649.50688907 h, G^{298} = -1649.456785 h, μ=3.63 D

Cartesian coordinates, Å

N	atom	x	y	z
1	C	-3.966087	0.753279	0.002359
2	C	-2.834225	-0.237620	-0.000431
3	O	-3.024420	-1.442507	-0.000256
4	C	-1.456878	0.343320	-0.003609
5	C	-0.387226	-0.446340	0.001971
6	C	1.038701	-0.008775	0.000223
7	Cl	1.274440	1.764989	-0.012672
8	Cl	1.846668	-0.711718	-1.463377
9	Cl	1.843934	-0.689949	1.475549
10	H	-0.504552	-1.527257	0.008704
11	H	-1.368894	1.423498	-0.009490
12	H	-4.923483	0.240396	0.007260
13	H	-3.885991	1.403933	0.876843
14	H	-3.893132	1.399828	-0.875846

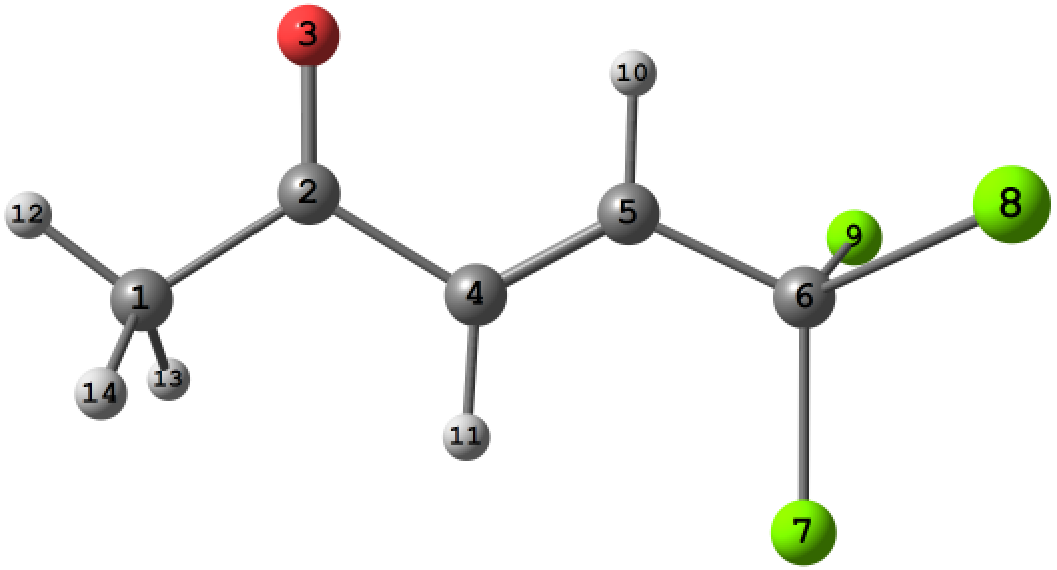

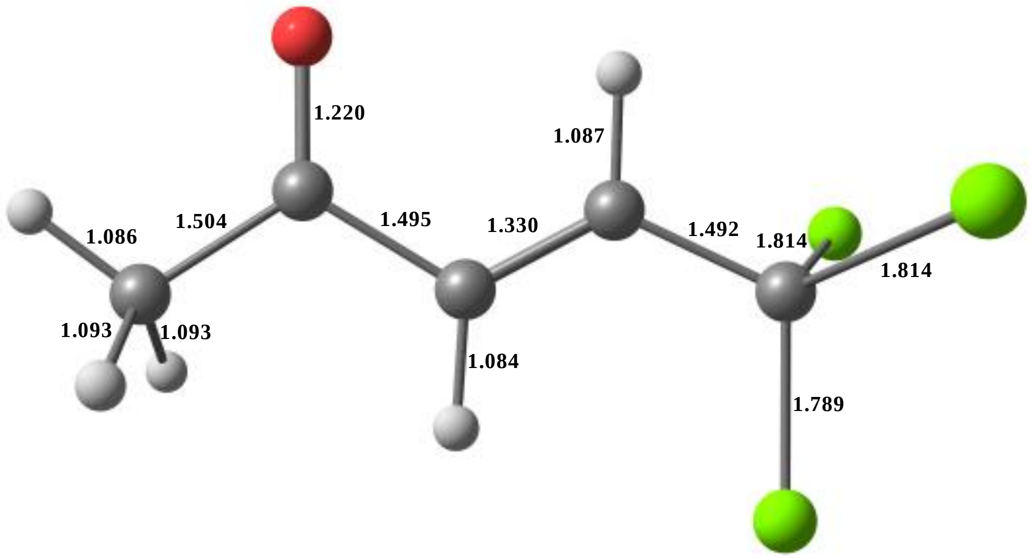

Summary of Natural Population Analysis:

Atom	No	Natural Charge	Natural Population Core	Valence	Rydberg	Total
C	1	-0.66767	1.99926	4.65730	0.01111	6.66767
C	2	0.56543	1.99936	3.40133	0.03388	5.43457
O	3	-0.61005	1.99975	6.58381	0.02649	8.61005
C	4	-0.26445	1.99890	4.24434	0.02122	6.26445
C	5	-0.18071	1.99881	4.15343	0.02848	6.18071
C	6	-0.16450	1.99903	4.10189	0.06357	6.16450
Cl	7	0.05009	9.99949	6.93023	0.02020	16.94991
Cl	8	0.03536	9.99952	6.94616	0.01896	16.96464
Cl	9	0.03536	9.99952	6.94616	0.01896	16.96464
H	10	0.26035	0.00000	0.73656	0.00310	0.73965
H	11	0.23982	0.00000	0.75793	0.00225	0.76018
H	12	0.22652	0.00000	0.77153	0.00195	0.77348
H	13	0.23716	0.00000	0.76143	0.00142	0.76284
H	14	0.23729	0.00000	0.76129	0.00142	0.76271
* Total *		0.00000	41.99362	51.75338	0.25299	94.00000

Cation A.
Energy E(B3LYP) = -1649.9211624 h, G^{298} = -1649.859621 h, μ=14.7 D

Cartesian coordinates, Å

N	atom	x	y	z
1	C	3.893739	0.826322	0.004235
2	C	2.755510	-0.112707	0.002776
3	O	2.908346	-1.380956	-0.000305
4	C	1.407918	0.398518	-0.002922
5	C	0.349866	-0.424597	0.005526
6	C	-1.085177	-0.003407	0.000778
7	Cl	-1.342734	1.759740	-0.017964
8	Cl	-1.839373	-0.705041	1.480507
9	Cl	-1.835642	-0.735618	-1.466437
10	H	0.474514	-1.505875	0.017547
11	H	1.300865	1.477033	-0.011834
12	H	4.852550	0.316339	0.062071
13	H	3.843750	1.428971	-0.908918
14	H	3.778841	1.522337	0.839835
15	H	3.853295	-1.720298	0.007586

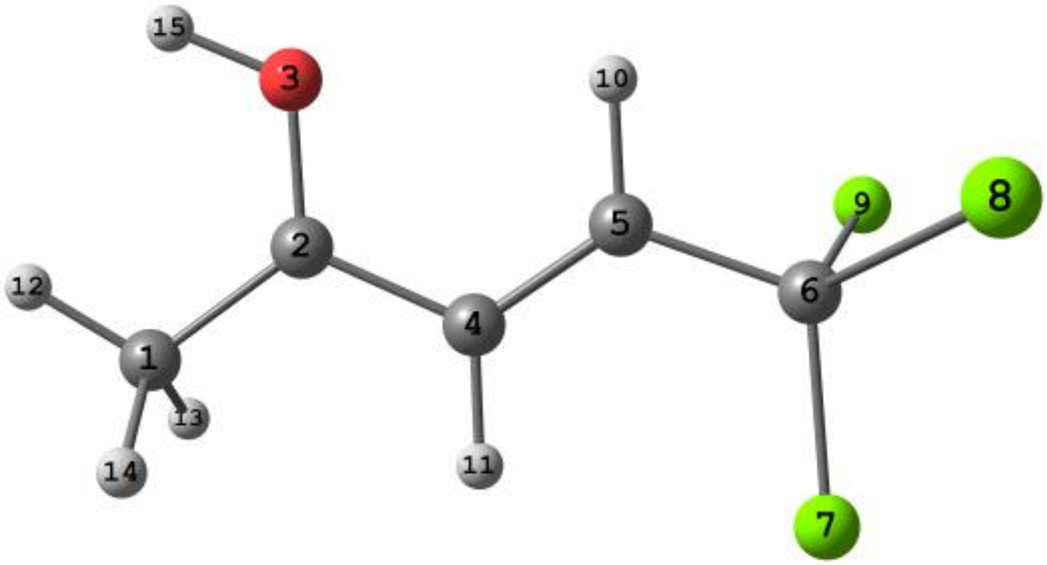

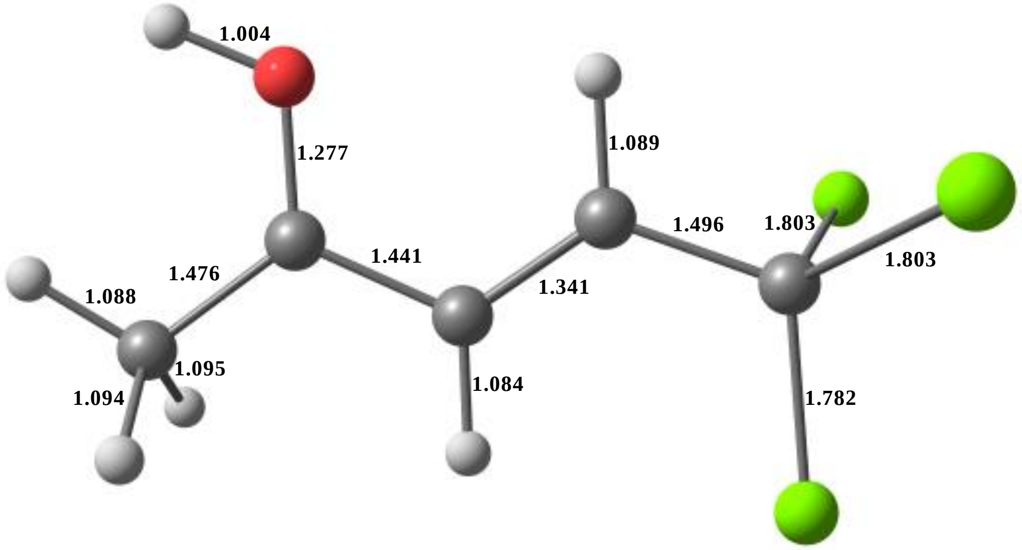

Summary of Natural Population Analysis:

Atom	No	Natural Charge	Core	Valence	Rydberg	Total
C	1	-0.67641	1.99921	4.66376	0.01343	6.67641
C	2	0.65535	1.99919	3.32172	0.02373	5.34465
O	3	-0.57363	1.99966	6.54829	0.02568	8.57363
C	4	-0.30672	1.99889	4.28552	0.02231	6.30672
C	5	-0.05936	1.99885	4.03219	0.02832	6.05936
C	6	-0.19233	1.99905	4.12769	0.06560	6.19233
Cl	7	0.07286	9.99949	6.90736	0.02029	16.92714
Cl	8	0.07171	9.99951	6.90957	0.01921	16.92829
Cl	9	0.07169	9.99951	6.90961	0.01919	16.92831
H	10	0.27474	0.00000	0.72246	0.00280	0.72526
H	11	0.27219	0.00000	0.72571	0.00209	0.72781
H	12	0.24646	0.00000	0.75155	0.00199	0.75354
H	13	0.28120	0.00000	0.71752	0.00127	0.71880
H	14	0.27719	0.00000	0.72153	0.00128	0.72281
H	15	0.58503	0.00000	0.41261	0.00237	0.41497
* Total *		1.00000	41.99336	51.75709	0.24954	94.00000

Core, Valence, Rydberg and Total are under the heading "Natural Population".

Cation B1.
Energy E(B3LYP) = -1650.28597516 h, G^{298} = - 1650.212865 h, μ=9.22 D

Cartesian coordinates, Å

N	atom	x	y	z
1	C	-3.638290	1.084646	-0.215506
2	C	-2.495766	0.171806	-0.365138
3	C	-1.113789	0.696968	-0.203672
4	C	-0.015090	-0.354973	-0.206162
5	Cl	0.011596	-1.328726	1.331365
6	O	-2.605136	-1.058482	-0.615788
7	C	1.383274	0.191802	-0.224078
8	Cl	2.554742	-0.642318	-1.023505
9	Cl	1.761542	1.592153	0.560727
10	H	-0.122210	-1.071447	-1.024891
11	H	-0.961551	1.386594	-1.051027
12	H	-4.547227	0.674603	-0.652194
13	H	-3.791491	1.222808	0.864060
14	H	-3.401276	2.064906	-0.629992
15	H	-1.092226	1.325701	0.693529
16	H	-3.538927	-1.435654	-0.751830

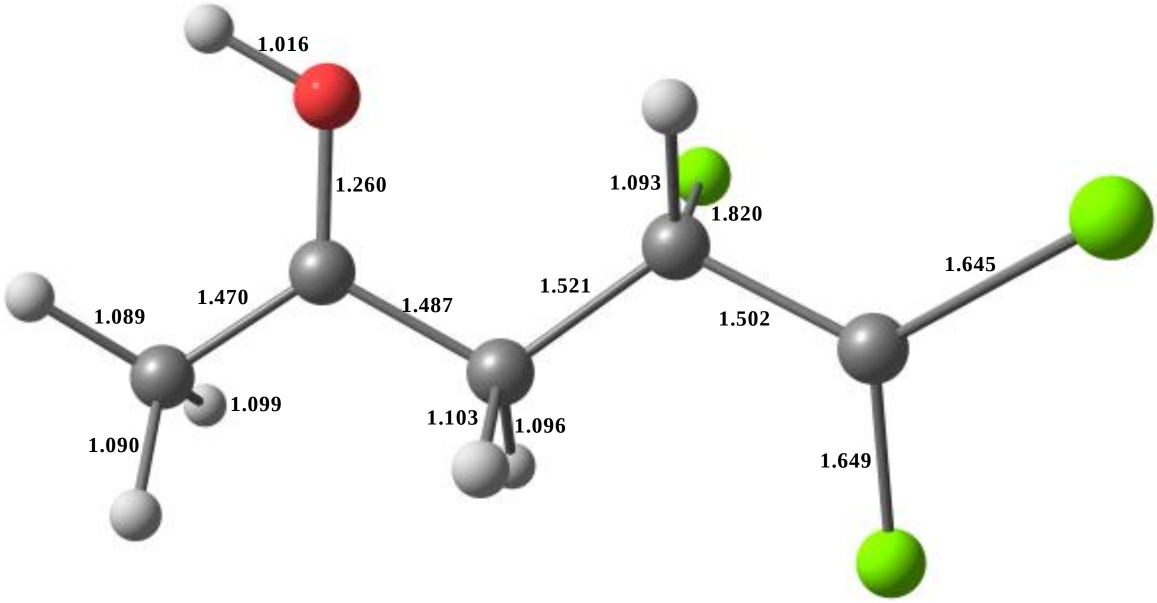

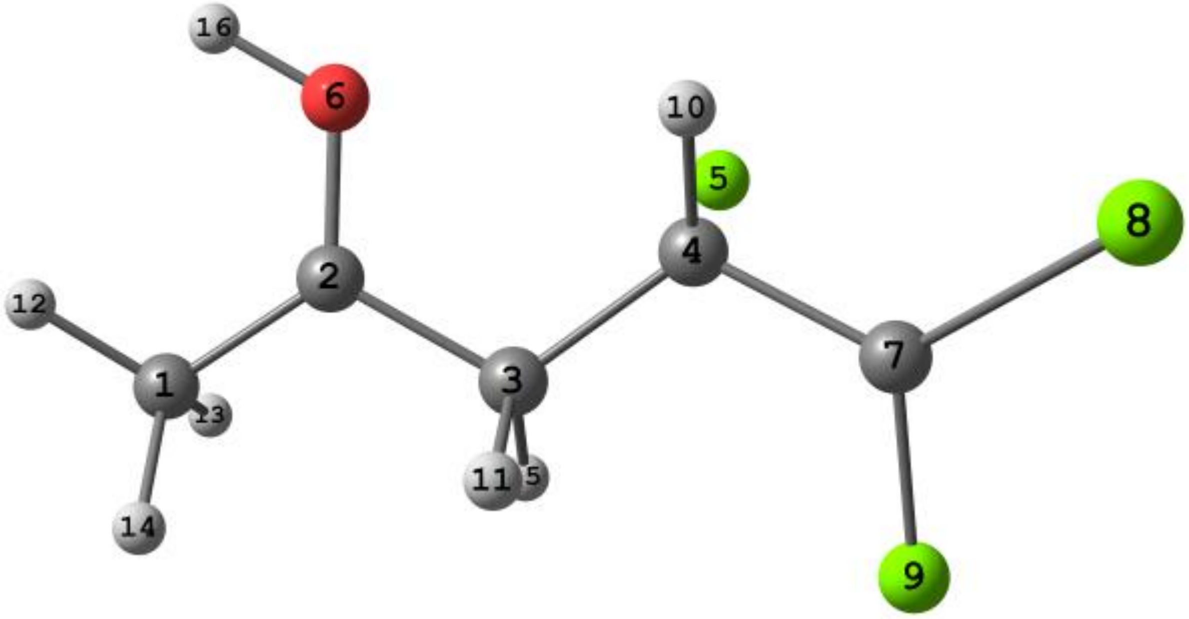

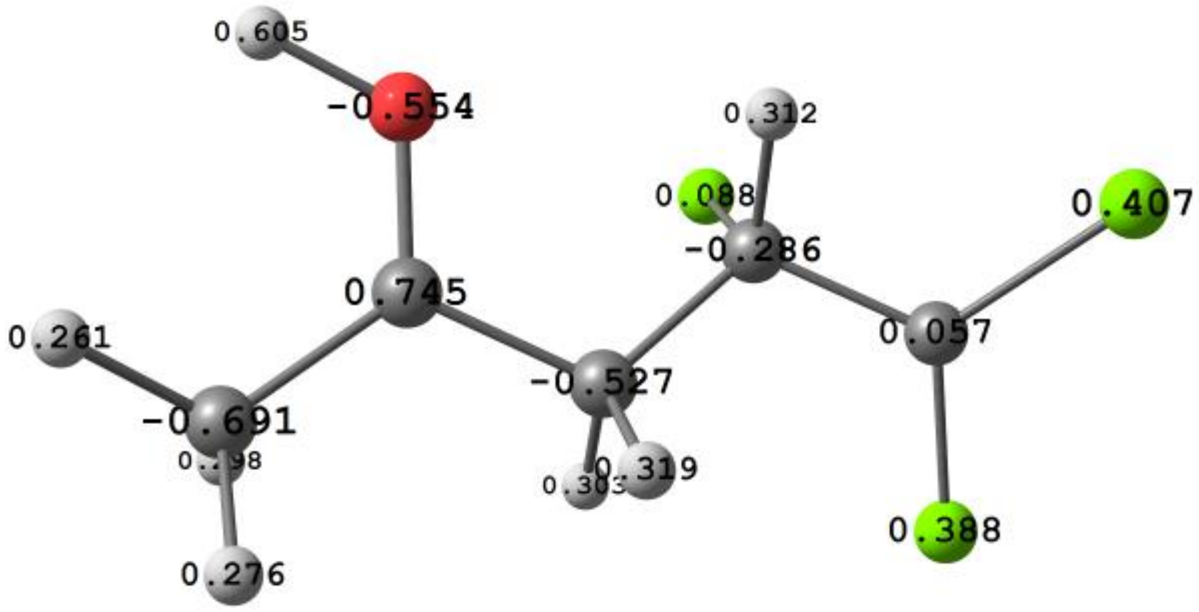

Summary of Natural Population Analysis:

Atom	No	Natural Charge	Natural Population: Core	Valence	Rydberg	Total
C	1	-0.69135	1.99919	4.67719	0.01497	6.69135
C	2	0.74464	1.99926	3.23135	0.02475	5.25536
C	3	-0.52707	1.99903	4.50432	0.02373	6.52707
C	4	-0.28598	1.99874	4.24839	0.03884	6.28598
Cl	5	0.08806	9.99969	6.89617	0.01609	16.91194
O	6	-0.55411	1.99964	6.52739	0.02708	8.55411
C	7	0.05749	1.99907	3.88976	0.05367	5.94251
Cl	8	0.40653	9.99917	6.56171	0.03259	16.59347
Cl	9	0.38805	9.99917	6.58036	0.03242	16.61195
H	10	0.31176	0.00000	0.68524	0.00300	0.68824
H	11	0.31897	0.00000	0.67955	0.00148	0.68103

H	12	0.26138	0.00000	0.73668	0.00194	0.73862
H	13	0.29824	0.00000	0.70041	0.00136	0.70176
H	14	0.27573	0.00000	0.72296	0.00130	0.72427
H	15	0.30290	0.00000	0.69522	0.00188	0.69710
H	16	0.60476	0.00000	0.39296	0.00228	0.39524

===

* Total *	2.00000	41.99296	51.72965	0.27739	94.00000

Cation C.
Energy E(B3LYP) = -1650.21256999 h, G^{298} = -1650.147312 h, μ=22.2 D

Cartesian coordinates, Å

N	atom	x	y	z
1	C	3.823542	-0.882913	0.160518
2	C	2.822615	0.155787	-0.038204
3	O	2.971692	1.393239	-0.016327
4	C	1.402393	-0.284984	-0.293594
5	C	0.317944	0.488239	0.123719
6	C	-1.129450	0.007295	0.018450
7	Cl	-1.482983	-0.929965	1.498080
8	Cl	-1.395165	-0.984197	-1.435871
9	Cl	-2.156736	1.473578	-0.046495
10	H	0.560738	1.226806	-0.761266
11	H	1.276978	-1.217675	-0.871677
12	H	4.776236	-0.478418	0.495719
13	H	3.437007	-1.639777	0.859940
14	H	3.926085	-1.411645	-0.802556
15	H	3.922668	1.782692	0.154955
16	H	0.497501	1.181494	0.963015

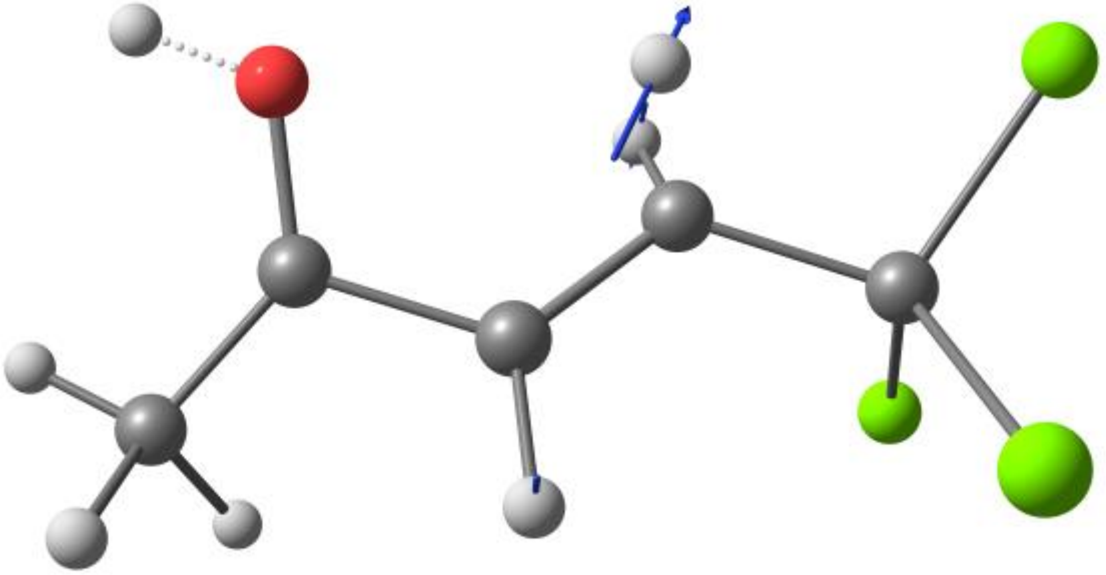

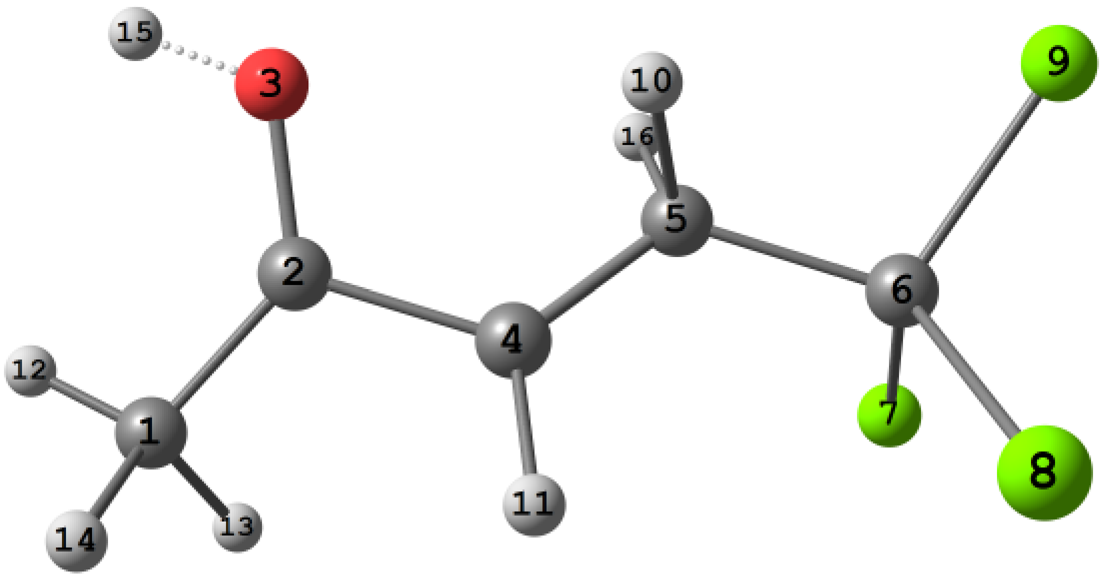

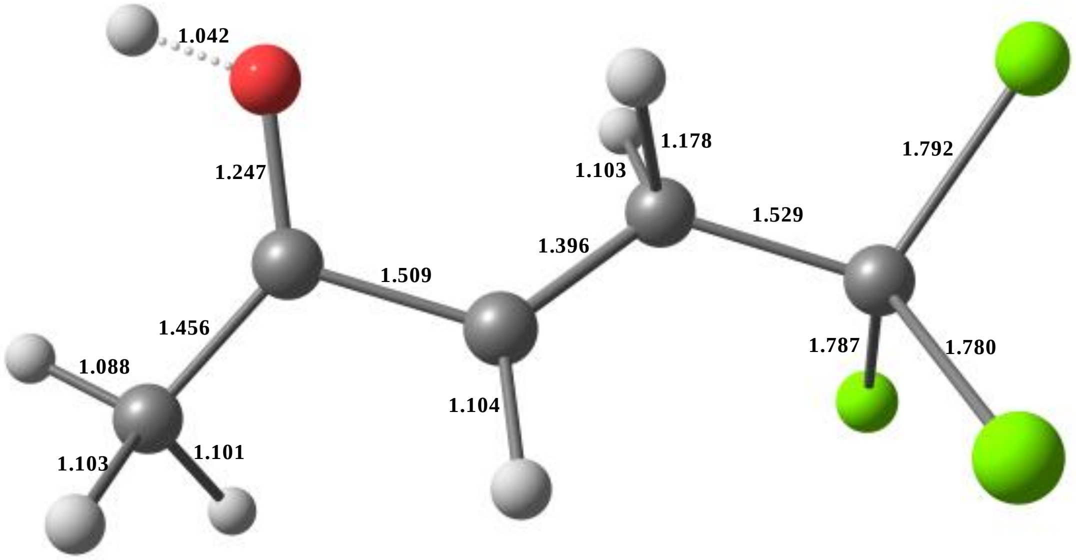

Summary of Natural Population Analysis:

Atom	No	Natural Charge	Core	Valence	Rydberg	Total
			Natural Population			
C	1	-0.69087	1.99919	4.67610	0.01558	6.69087
C	2	0.64636	1.99923	3.32980	0.02461	5.35364
O	3	-0.49958	1.99963	6.47469	0.02525	8.49958
C	4	0.25581	1.99905	3.72456	0.02058	5.74419

C	5	-0.56420	1.99888	4.52788	0.03743	6.56420
C	6	-0.18546	1.99916	4.11556	0.07074	6.18546
Cl	7	0.11120	9.99948	6.86904	0.02028	16.88880
Cl	8	0.09747	9.99948	6.88249	0.02056	16.90253
Cl	9	0.10189	9.99950	6.87852	0.02010	16.89811
H	10	0.47791	0.00000	0.51831	0.00378	0.52209
H	11	0.32781	0.00000	0.66952	0.00268	0.67219
H	12	0.28329	0.00000	0.71522	0.00149	0.71671
H	13	0.30966	0.00000	0.68912	0.00122	0.69034
H	14	0.32072	0.00000	0.67811	0.00118	0.67928
H	15	0.64409	0.00000	0.35380	0.00211	0.35591
H	16	0.36390	0.00000	0.63345	0.00266	0.63610
* Total *		2.00000	41.99360	51.73614	0.27025	94.00000
